# Global Transcriptional Analysis of Spontaneous Sakacin P-Resistant Mutant Strains of *Listeria monocytogenes* during Growth on Different Sugars

**DOI:** 10.1371/journal.pone.0016192

**Published:** 2011-01-06

**Authors:** Girum Tadesse Tessema, Trond Møretrø, Lars Snipen, Lars Axelsson, Kristine Naterstad

**Affiliations:** 1 Nofima Mat AS, Ås, Norway; 2 Department of Chemistry, Biotechnology and Food Science, Norwegian University of Life Sciences, Ås, Norway; Queen Mary University of London, United Kingdom

## Abstract

Subclass IIa bacteriocins have strong antilisterial activity and can control the growth of *Listeria monocytogenes* in food. However, *L. monocytogenes* may develop resistance towards such bacteriocins. In this follow-up study, the transcriptomes of a high level (L502-1) and a low level (L502-6) spontaneous sakacin P-resistant mutant strain of *L. monocytogenes* were compared to the wild-type (L502). The growth of the resistant strains was reduced on mannose but not affected on cellobiose and the transcriptomics was performed during growth on these sugars. The mannose phosphotransferase system (PTS) encoded by the *mptACD* operon (*mpt*) is known for transporting mannose and also act as a receptor to class IIa bacteriocins. The *mpt* was repressed in L502-1 and this is in accordance with abolition of the bacteriocin receptor with resistance to class IIa bacteriocins. In contrast, the *mpt* was induced in L502-6. Despite the induction of the *mpt*, L502-6 showed 1,000 times more resistance phenotype and reduced growth on mannose suggesting the mannose-PTS may not be functional in L502-6. The microarray data suggests the presence of other transcriptional responses that may be linked to the sakacin P resistance phenotype particularly in L502-6. Most of commonly regulated genes encode proteins involved in transport and energy metabolism. The resistant strains displayed shift in general carbon catabolite control possibly mediated by the *mpt*. Our data suggest that the resistant strains may have a reduced virulence potential. Growth sugar- and mutant-specific responses were also revealed. The two resistant strains also displayed difference in stability of the sakacin P resistance phenotype, growth in the presence of both the lytic bacteriophage P100 and activated charcoal. Taken together, the present study showed that a single time exposure to the class IIa bacteriocin sakacin P may elicit contrasting phenotypic and transcriptome responses in *L. monocytogenes* possibly through regulation of the *mpt*.

## Introduction

Listeriosis is a rare but potentially serious infectious disease. Consumption of food contaminated with *Listeria monocytogenes* is the major route of transmission [1]. In recent years, a resurgent trend in listeriosis has been reported in many countries [2]. *L. monocytogenes* is also responsible for substantial food product recalls and considerable economic loss to the food industry [3].

Bacteriocins produced by lactic acid bacteria are of major interest as natural and safe food preservatives targeting foodborne pathogens, including *L. monocytogenes* [4]–[7]. The lactic acid bacteria bacteriocins are grouped into several classes and subclasses [7]. Subclass IIa bacteriocins (also called pediocin-like) are considered the most important class II bacteriocins and have strong antilisterial activity [8].

Development of resistance by target cells after exposure to class IIa bacteriocins seriously hampers the use of these bacteriocins as biopreservatives [9], [10]. The molecular mechanism behind the resistance is not fully known and is important to elucidate for optimal use of the bacteriocins. In *L. monocytogenes* and other related gram positive bacteria, it is established that the mannose specific phosphotransferase system (PTS) encoded by the *mptACD* operon (hereafter *mpt*) is the receptor for class IIa bacteriocins, and abolition of the receptor is the most common mechanism behind resistance to class IIa bacteriocins [10]–[17]. A more recent transcriptome study [18] on class IIa bacteriocin-resistant *Enterococcus faecalis* indicated that, in addition to conferring resistance to class IIa bacteriocins, the mannose-PTS plays a key role in global carbon catabolite control. Interestingly, up-regulation of the *mpt* among class IIa bacteriocin-resistant mutants has been reported [19], [20].

The virulence potential of class IIa bacteriocin-resistant strains is a concern that has to be addressed. A study by Gravesen et al. has investigated the expression of 13 selected virulence genes for two class IIa bacteriocin-resistant strains using targeted microarray (64 genes) and the study has shown that five of the virulence genes were significantly down-regulated in one of the resistant strains [19]. For the other resistant strain, the authors has reported a non-significant induction in the tested virulence genes [19]. In another study, exposing of *L. monocytogenes* to a sakacin 1 producing strain [21] showed no apparent effect of this class IIa bacteriocin on the hemolytic activity of *L. monocytogenes* [22]. Hence, more study is needed to have a clearer understanding on the relationship between class IIa bacteriocin resistance with virulence.

We have recently shown that based on the IC_50_ (50% inhibitory concentration) of sakacin P, spontaneous sakacin P-resistant mutants of *L. monocytogenes* could be grouped into strains with high levels of resistance (IC_50_, ≥10 µg ml^−1^) and strains with low levels of resistance (IC_50_, <10 µg ml^−1^) [20]. The present study is a continuation of the previous work [20], in which a representative strain from each level of resistance group of *L. monocytogenes* L502 were chosen for further in-depth characterization. The high level resistant strain *L. monocytogenes* L502-1 (hereafter L502-1) shows a slight growth reduction on mannose, with down-regulation of the bacteriocin receptor gene (*mptA*), compared to that of the wild-type L502. In contrast, the low level sakacin P-resistant *L. monocytogenes* L502-6 (hereafter L502-6) shows dramatic growth reduction on mannose, and the *mptA* gene is up-regulated. The two resistant strains grow as the wild-type on cellobiose [20]. The observed high expression of the bacteriocin receptor gene (*mpt*) in two class IIa bacteriocin-resistant strains from our previous work [20] and from another study by Gravesen et al. [19] is intriguing and requires further investigation.

The main purpose of the present study was to identify possible genes that might be involved in sakacin P resistance particularly in the mutant strain that showed induction of the bacteriocin receptor. Genome-wide transcriptome profile of the two different sakacin P-resistant strains (L502-1 and L502-6) were compared to that of the wild-type (L502) upon growth on mannose or cellobiose. The data presented here independently confirm our previous report regarding with the repression and induction of the *mpt* operon in L502-1 and L502-6 respectively. Nevertheless, the transcriptomic and phenotypic results altogether suggest the *mpt* operon could be involved in the sakacin P resistance not only in L502-1 but also in L502-6. In addition, the transcriptomics suggests the presence of other transcriptional responses that may be linked to the sakacin P resistance in L502-6. To our knowledge, the present study reports the first genome-wide transcriptome profiling in spontaneous class IIa bacteriocin-resistant mutant strains of *L. monocytogenes* and opens new possibilities for further studies.

## Results and Discussion

The class IIa bacteriocins can inhibit unwanted microorganisms in food including the foodborne pathogen *L. monocytogenes* [23]. Development of spontaneous mutant strains with reduced susceptibility to the bacteriocin creates significant concern with regard to application of the bacteriocins as food preservative [24]. A deeper knowledge about the acquired resistance is crucial for optimal use of the bacteriocins in the food industry. Recently we have shown that substantial diversity among large number of *L. monocytogenes* spontaneous mutant strains obtained after a single time exposure to the class IIa bacteriocin sakacin P [20]. The present study is a continuation of the previous work [20], in which two different sakacin P-resistant strains of *L. monocytogenes* L502 were further characterized mainly using whole-genome DNA microarray.

### Global gene expression profile and validation by quantitative real-time reverse transcriptase PCR

**Figure 1:**
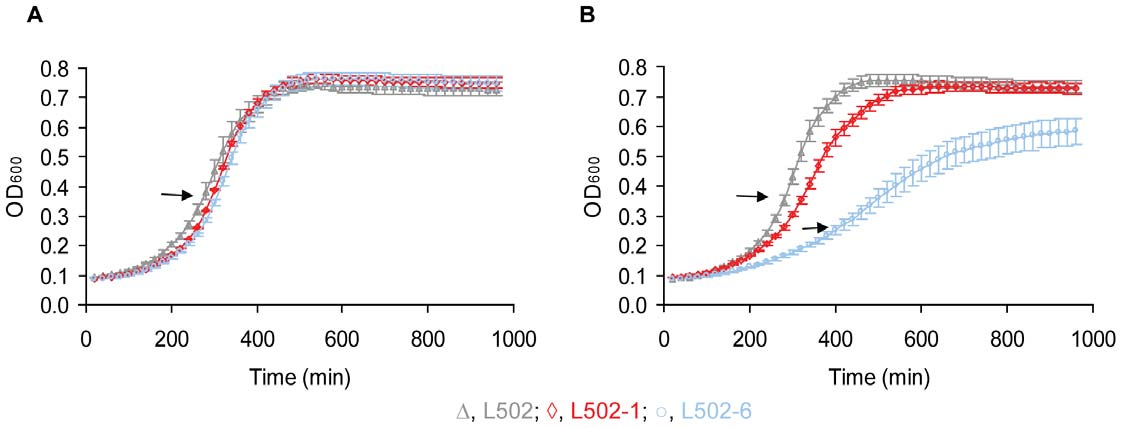
Growth of *L. monocytogenes* L502 and its sakacin P-resistant strains. (**A**) growth on cellobiose and (**B**) growth on mannose. The solid arrows indicate time of harvesting. The error bars represent standard errors of the mean.

**Figure 2:**
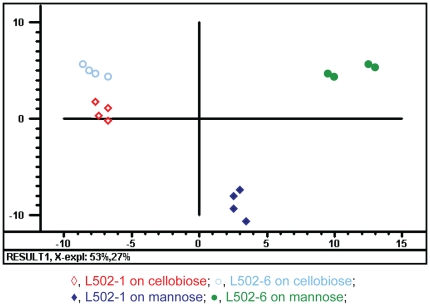
PCA score plot of the differentially regulated genes in the sakacin P-resistant strains. PCA score plot displaying global transcriptome profiles of L502-1 and L502-6 grown on mannose or on cellobiose compared to the wild-type strain grown on the same sugar. The two biological samples and their dye-swap replicates were included in the PCA. The explained variances for the two first components; PC1and PC2 are 53% and 27% respectively.

In the present study we compared the transcriptome profiles of two different spontaneous sakacin P-resistant mutant strains of *L. monocytogenes* with their wild-type strain (Table 1). The growth of the resistant strains was reduced on mannose but the resistance strains grow as wild-type on cellobiose (Table 1, Figure 1, [20]). For better understanding of the incidence(s) giving rise to the sakacin P resistance (i.e. resistance conferring mutation), the transcriptome analysis was performed during growth on mannose or cellobiose. In the present study, the transcriptome of the sakacin P-resistant mutant strains grown on mannose or cellobiose were compared to the wild-type strain grown on the same sugar (e.g. resistant strain on mannose/wild-type strain on mannose). During growth on mannose a total of 124 (87 up and 37 down) and 194 (152 up and 42 down) genes were regulated in L502-1 and L502-6 respectively, compared to the wild-type. The total number of genes affected on cellobiose were 39 (20 up and 19 down) in L502-1 and 36 (30 up and 6 down) in L502-6. The complete list of the differentially regulated genes in any of the growth conditions is available in Table S1. The result from Principal Component Analysis (PCA) indicates main variation in global transcriptional response was due to growth sugar effect followed by difference between the two resistant strains (Figure 2). These overall global transcriptional responses of the resistant strains grown on mannose and cellobiose was in line with the fitness of the resistant strains growing on the respective sugars (Figure 1, Table 1, [20]).

**Table 1:** The sakacin P-resistant strains of *L. monocytogenes* L502 used in this study.

	Characteristics^a^			
Sakacin P resistant strains	Level of resistance to sakacin P	Growth rate on mannose (%)	Growth rate on cellobiose (%)	*mptA* gene expression
L502-1	>10^6^	82	107	repressed
L502-6	10^3^	44	105	induced

a relative to the wild-type strain (mutant/wild-type) and detail can be found in reference [20].

Analysis of the differentially expressed genes according to the Comprehensive Microbial Resource of the J. Craig Venter Institute (CMR-JCVI) *L. monocytogenes* EGDe genome database role categories showed that the sakacin P-resistant strains had altered expression of genes belonging to the majority of the role categories (Figure S1 and Table S1). This indicates that the incidence(s) giving rise to the sakacin P resistance also affects the overall physiology of the resistant strains, which is in agreement with our earlier finding on intact cell profile of the sakacin P-resistant strains using Fourier transform infrared spectroscopy [20]. In general, genes belonging to the energy metabolism, transport and binding proteins, and amino acid biosynthesis of the CMR-JCVI role categories were overrepresented (p<0.001 and odd ratios >2) and most of them were induced (Figure S1).

The reliability of the microarray expression data was assessed by quantitative real-time reverse transcriptase PCR (qRT-PCR) analysis. The validation assay showed strong correlation of gene expression with the microarray results under all tested conditions (r>0.9, Figure S2), confirming independently the reliability of the microarray data. The microarray probes used in the present study were based on the EGDe strain (Accession No. A-BUGS-19; http://bugs.sgul.ac.uk/A-BUGS-19). More than 94% of *L. monocytogenes* L502 genes were hybridized to the EGD-e based probes in any of experimental conditions tested so far (not shown). A recent study [25] has shown that the EGDe strain expresses comparable number of its genes (>98% of its open reading frames) under different growth conditions. Taken together, the microarray analysis reported in the present study unambiguously reflects the global transcriptomic profiles of the sakacin P-resistant strains.

### Transcriptional responses linked to resistance to sakacin P

The sakacin P-resistance phenotype was confirmed during growth on mannose and cellobiose using selective plates containing sakacin P at the time of harvesting. The growth fitness of the resistant strains was not affected in the presence of sakacin P when cultivated on mannose and cellobiose (not shown). Consequently, genes whose expression altered in both sugars were considered as potential genes that might be linked to the resistance. Genes differentially regulated in both sugars in L502-1 and L502-6 are listed in Table 2 and Table 3 respectively.

**Table 2:** Differentially regulated genes in L502-1 during growth on both sugars.

		Mannose^a^		Cellobiose	
Locus (gene)	Product (similar to)	Log2	q-value	Log2	q-value
lmo0027	PTS, beta-glucosides specific enzyme IIABC	1.5	10^−5^	4.1	10^−7^
lmo0096 (*mptA*)	PTS mannose-specific, factor IIAB	−2.3	10^−7^	1.4	10^−4^
lmo0097 (*mptC*)	PTS mannose-specific, factor IIC	−2.4	10^−9^	1.5	10^−6^
lmo0098 (*mptD*)	PTS mannose-specific, factor IID	−2.4	10^−11^	1.4	10^−7^
lmo0204 (*actA*)	actin-assembly inducing protein precursor	−0.7	10^−5^	−0.8	10^−4^
lmo0781 (*mpoD*)	mannose-specific PTS component IID	1.2	10^−6^	1	10^−7^
lmo0782 (*mpoC*)	mannose-specific PTS component IIC	1.4	10^−6^	1.7	10^−8^
lmo0783 (*mpoB*)	mannose-specific PTS component IIB	1.2	10^−6^	1.9	10^−7^
lmo0784 (*mpoA*)	mannose-specific PTS component IIA	1.5	10^−6^	2.1	10^−9^
lmo0848	amino acid ABC transporter, ATP-binding protein	0.6	10^−4^	0.7	10^−6^
lmo1251	regulator of the Fnr CRP family (including PrfA)	−1.4	10^−7^	−1.4	10^−6^
lmo1254	alpha, alpha-phosphotrehalase	2.3	10^−8^	0.6	10^−5^
lmo1255	PTS trehalose specific enzyme IIBC	2.5	10^−9^	0.6	10^−7^
lmo2250 (*arpJ*)	amino acid ABC transporter, permease protein	−0.7	10^−7^	−0.6	10^−3^
lmo2683	cellobiose PTS enzyme IIB component	−1.5	10^−6^	−1.4	10^−8^
lmo2684	cellobiose PTS enzyme IIC component	−1.7	10^−9^	−0.8	10^−6^
lmo2685	cellobiose PTS enzyme IIA component	−1.3	10^−6^	−1.4	10^−8^

a Differentially regulated genes in the high level sakacin P-resistant strain (L502-1) during growth on mannose and on cellobiose. Log2 (expression ratio) is relative to the wild-type strain and q-value is a p-value adjusted to control false discovery rate.

**Table 3:** Differentially regulated genes in L502-6 during growth on both sugars.

		Mannose^a^		Cellobiose	
Locus (gene)	Product (similar to)	Log2	q-value	Log2	q-value
lmo0096 (*mptA*)	PTS mannose-specific, factor IIAB	1.6	10^−7^	2.6	10^−7^
lmo0097 (*mptC*)	PTS mannose-specific, factor IIC	1.6	10^−6^	2.7	10^−7^
lmo0098 (*mptD*)	PTS mannose-specific, factor IID	1.6	10^−6^	2.6	10^−9^
lmo0099	unknown	1.5	10^−6^	0.8	10^−4^
lmo0361 (*tatC*)	twin arginine translocase C	1.2	10^−5^	1.2	10^−4^
lmo0362 (*tatA*)	twin arginine translocase A	0.9	10^−4^	1.0	10^−3^
lmo0365	conserved hypothetical protein	1.4	10^−4^	1.5	10^−4^
lmo0366	putative lipoprotein	1.1	10^−5^	1.2	10^−3^
lmo0367	*B. subtilis* YwbN protein	1.4	10^−5^	1.5	10^−4^
lmo0433 (*inlA*)	internalin A	−0.8	10^−6^	−0.6	10^−5^
lmo0485	unknown	0.6	10^−3^	0.8	10^−3^
lmo0541	ABC transporter (binding protein)	0.8	10^−3^	1.0	10^−3^
lmo0847	glutamine ABC transporter (binding and transport protein)	0.7	10^−5^	1.0	10^−5^
lmo0848	amino acid ABC transporter, ATP-binding protein	0.7	10^−5^	0.9	10^−4^
lmo1007	unknown	0.7	10^−4^	0.7	10^−3^
lmo1251	regulator of the Fnr CRP family (including PrfA)	−2.0	10^−6^	−1.6	10^−5^
lmo1566 (*citC*)	isocitrate dehyrogenases	0.7	10^−3^	0.7	10^−3^
lmo1567 (*citZ*)	citrate synthase subunit II	0.8	10^−5^	0.8	10^−4^
lmo1641	aconitate hydratases	0.8	10^−4^	0.9	10^−5^
lmo1957 (*fhuG*)	ferrichrome ABC transporter (permease)	0.6	10^−4^	0.7	10^−3^
lmo1958 (*fhuB*)	ferrichrome ABC transporter (permease)	0.6	10^−4^	0.7	10^−3^
lmo1959 (*fhuD*)	ferrichrome binding protein	0.8	10^−5^	1.0	10^−3^
lmo1960 (*fhuC*)	ferrichrome ABC transporter (ATP-binding protein)	0.6	10^−4^	0.6	10^−3^
lmo2181 (*srtB*)	Sortase B protein	0.9	10^−4^	1.0	10^−3^
lmo2182	ferrichrome ABC transporter (ATP-binding protein)	0.9	10^−4^	1.1	10^−3^
lmo2183	ferrichrome ABC transporter (permease)	0.9	10^−5^	1.0	10^−3^
lmo2184	ferrichrome ABC transporter (binding protein)	1.2	10^−4^	1.2	10^−4^
lmo2185 *(svpA)*	surface virulence-associated protein, substrate for SrtB	1.1	10^−4^	1.2	10^−3^
lmo2186 *(isdC)*	iron-regulated surface determinants, substrate for SrtB	1.3	10^−4^	1.4	10^−4^
lmo2363 (*gadD2*)	glutamate decarboxylase	−0.8	10^−3^	−1.1	10^−3^
lmo2781	beta-glucosidase	0.8	10^−6^	0.6	10^−5^

a Differentially regulated genes in the low level sakacin P-resistant strain (L502-6) during growth on mannose and on cellobiose. Log2 (expression ratio) is relative to the wild-type strain and q-value is a p-value adjusted to control false discovery rate.

#### I) Abolition of the bacteriocin receptor (mpt)

In *L. monocytogenes*, it has been shown that the gene product of *mpt* is necessary for growth on mannose [26] and act as a receptor for the bacteriocin sakacin P [17], [27]. The transcriptome assay showed that during growth on mannose the *mpt* was down-regulated in the high level sakacin P-resistant strain (L502-1) compared to the wild-type (Table 2). In contrast, under similar growth condition, the *mpt* was up-regulated in the low level sakacin P-resistant strain (L502-6) (Table 3). In both sakacin P-resistant strains the *mpt* was up-regulated during growth on cellobiose (Table 2 and Table 3). To verify this, qRT-PCR assay was performed on *mptA* gene representing the *mpt* operon to investigate if cellobiose induces the *mpt* among the resistant strains. No significant induction of the *mptA* gene was observed in any of the strains during growth on cellobiose. In contrast, mannose induced the *mptA* gene in the following increasing order: L502-6>wild-type>L502-1 (Figure S3). Previously it has been shown that in wild-type *L. monocytogenes* strain the *mpt* gene is induced by the presence of mannose but not by cellobiose [11], [26].

The repression of *mpt* in L502-1 reported in the present study suggests that repression of the *mpt* could be a part of sakacin P resistance (Table 2). Indeed, several seminal studies uncovered the role of mannose-PTS in resistance/susceptibility of target cells for the class IIa bacteriocin [10]–[15], [17]–[19], [27], [28]. The induction of the *mpt* gene which encodes the bacteriocin receptor in L502-6 was intriguing (Table 3). Previously we have shown that another low level sakacin P-resistant strain (L40-6) also displayed induction of the *mptA* gene [20]. A similar trend in induction of the bacteriocin receptor in a carnobacteriocin B2-resistant strain at transcript and protein levels has been reported [19]. Despite the induction of the *mpt*, the two low level resistant strains studied in our laboratory showed 1,000 times more resistance phenotype than their respective wild-type strains. In addition, the growth of these resistant strains was reduced significantly particularly on mannose (Table 1). This may suggests the normal function of the gene product of *mpt* is affected not only in L502-1 but also in L502-6. Hence, repression of the receptor (decrease in expression or modification) could be the main resistance mechanism against the sakacin P in both resistant strains. Nevertheless, how the induced *mpt* expression in L502-6 confers resistance to sakacin P is not clear and requires further investigation. The transcriptome data also suggest the presence of other transcriptional responses that may contribute to the sakacin P-resistance phenotype particularly in L502-6 (see below).

Genes known to modulate the *mpt* expression and conferring resistance to a class IIa bacteriocin were not differentially expressed in any of the sakacin P-resistant strains when compared to the wild-type. This is in contrast to previous reports on defined mutants lacking functional σ^54^ factor [29], ResD (two-component response regulator) [30], Lmo0095 (*mpt* activator) [31], or PrfA (positive regulatory factor A) [19], and having increased resistance to class IIa bacteriocin possibly through inactivation of the mannose PTS. Deletion of the *manR* gene (encoding transcriptional activator for σ ^54^) resulted in resistance to a class IIa bacteriocin [11]. In the present study and in other spontaneous class IIa bacteriocin-resistant mutants studied [19], the expression of *manR* was generally up-regulated in the resistant strains compared to the wild-type (Table S1). The role of ManR in spontaneous class IIa bacteriocin resistance remains to be studied. In general, the *pts* genes are known to be affected by factors as type of strain used (defined versus spontaneous mutants) and the growth conditions (e.g. defined culture media versus complex media) [19], [26], [32].

#### II) Modification of the cell envelope and antimicrobial translocation system

Genes encoding proteins associated with the cell envelope were induced in L502-6 (Table 3 and Table S1). This includes *srtB* which encodes a sortase protein that modifies the cell envelope and *lmo0366* encoding a putative surface lipoprotein (Table 3). The link between modification of the cell envelope and resistance to class IIa bacteriocins reported previously [15], [33], [34]. In the present study, *tatAC* operon encoding the Twin-Arginine Translocase (TAT) system was up-regulated in L502-6 (Table 3). In other bacteria as *Mycobacterium smegmatis* and *Campylobacter jejuni*, mutation of the *tat* specific genes increased susceptibility to antimicrobials indicating the TAT dependent translocation of antimicrobials [35], [36]. Our transcriptional data together with results from the previous studies may suggest modification of the cell envelop and the TAT system may contribute to the sakacin P-resistance phenotype in strain L502-6. However, it is yet unclear whether these may also represent an indirect consequence of the altered *mpt* expression (see below).

### The *mpt* could be involved in global carbon catabolite control

In the present study genes associated with energy transport and metabolism were the most affected in the resistant strains particularly upon growth on mannose (p<0.001 and odd ratios >2, Figure S1). *In-silico* analysis for catabolite responsive element (*cre*) sequence (WWTGNAARCGNWWWCAWW) described for *Bacillus subtilis* [37] indicated that a number of the differentially regulated genes harbor potential *cre* sites important for carbon catabolite control via catabolite control protein A (CcpA) (not shown). Among the genes with potential *cre* site a number of them are previously described to be under carbon catabolite control in *L. monocytogenes* [31], [38], [39] and the closely related bacteria *B. subtilis* (reviewed in [40]).

Known genes that are under catabolite control repression (CCR) and were induced in the resistant strains includes *lmo0027* (encode β-glucosides specific permase) [31], *lmo1254/lmo1255* (trehalose metabolism) [40], *glpK* and *glp*D (glycerol metabolism) [40], *citC*, *citZ* and *citB* (oxidation portion of tricarboxylic acid cycle) [39], [40], and *lmo0847* (a putative glutamine ATP-binding cassette (ABC) transporter) [39]. In addition a number of *pts* and other transporter and utilization genes for sugars that was not present in the growth media (e.g. fructose, galacitol, ascorbate, maltose and manitol) and their transcriptional activators and antiterminators were induced (Table S1). Conversely, genes *alsS* and *alsD* which involved in acetoin biosynthesis are under carbon catabolite activation [40] and here were found to be down-regulated in the resistant strains (Table S1). The transcriptome data indicated that none of the genes encoding proteins mediating carbon catabolite control (e.g. *ptsI*, *hprK*, *ccpA*, *ccpC*, and *cggr*) were differentially regulated (not shown).

The majority of the transcriptome changes observed in the present study could be linked to global carbon catabolite control mediated by the altered *mpt* as shown in previous studies [18], [26], [31], [41]. In addition to a role in carbon catabolite control, the mannose specific PTS is involved in a variety of cellular functions including, stress tolerance [42], biofilm formation [41] and virulence [31]. In fact, the sakacin P-resistant strains studied in the present study displayed low tolerance to food related stress [20], produce less biofilm [20] and may have reduced virulence potential (see below) compared to the wild-type. Therefore, it is possible that the regulatory role of the *mpt* affected other genes including a number of known and putative genes encoding regulatory proteins generally associated with the PTSs (Table S1) [43]. Further studies will be required to fully define the role of the *mpt* in *L. monocytogenes*.

### Common and specific transcriptional response to the high- and low-level resistant strains

**Figure 3:**
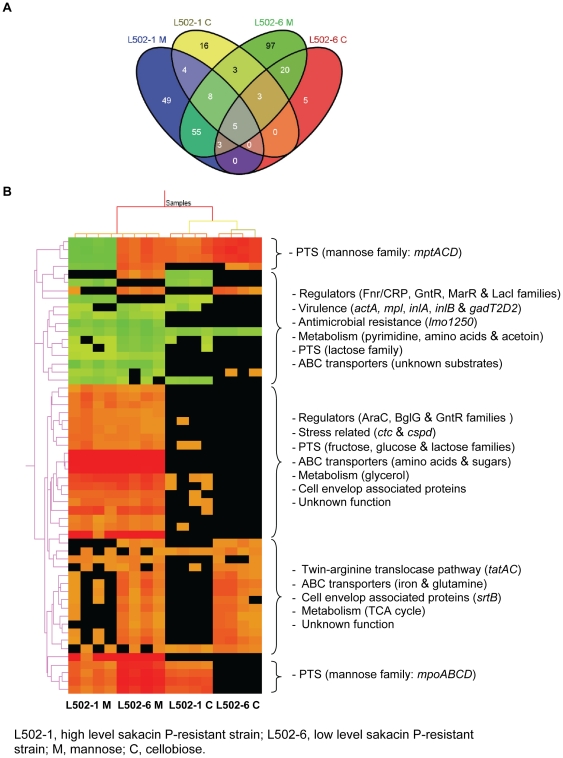
Presentation of the differentially regulated genes using a Venn diagram and heatmap. (**A**) A Venn diagram showing total number of common and specific genes differentially regulated in the sakacin P-resistant strains (L502-1 and L502-6) grown on mannose (M) or cellobiose (C) compared to the wild-type strain grown on the respective sugar. (**B**) A heatmap showing the differentially regulated genes in the sakacin P-resistant strains (L502-1 and L502-6) during growth on mannose (M) or on cellobiose (C) compared to the wild-type strain (L502) grown on the respective sugar. The designated biological role(s) of proteins encoded by the genes with similar pattern of regulations are shown in bracket. Color code: red, up-regulated; green, down-regulated; black, not differentially expressed.

As it can be seen in Figure 3, common and unique genes were differentially expressed in the high level and the low level sakacin P-resistant strains upon growth on mannose and on cellobiose. Commonly down-regulated genes in the resistant strains during on mannose include genes ascribed in virulence, antimicrobial resistance, pyrimidine metabolism and amino acid biosynthesis (Figure 3B, Table S1). The reduced expression of genes encoding pyrimidine and amino acid metabolism upon growth on mannose is inline with the growth fitness of the resistant strains on this sugar (Figure 1, Table 1). As mentioned earlier, genes encoding proteins associated with energy transport and metabolism were commonly up-regulated in the sakacin P-resistant strains during growth on mannose (p<0.001 and odd ratios >2, Figure 3B, Table S1).

The common genes up-regulated and down-regulated under all tested conditions were *lmo0848* and *lmo1251* respectively. The operon encoded by *lmo0847*and *lmo0848* is an ABC glutamine transporter and as mentioned earlier it is known to be under CCR [39]. The *lmo1251* is a PrfA-like putative CRP-FNR (cyclic AMP receptor protein-fumarate and nitrate reduction) family transcriptional regulator and the CRP-FNR families mainly functions as positive transcriptional regulators and are known to control genes in various aspects as sugar and amino acid transport and metabolism and pathogenesis [44].

A total of 49 genes (33 up-regulated and 16 down-regulated) were affected only in L502-1 upon growth on mannose (Figure 3A, Table S1). A number of genes differentially regulated in L502-1 were associated with transport and utilization of energy (p<0.001 and odd ratios >2). In contrast, upon growth on mannose large number of genes (69 up-regulated and 28 down-regulated) encoding proteins associated with different cellular functions were differentially regulated in L502-6. In addition many genes encoding unknown proteins were induced in L2502-6 (Figure S1, Table S1). Despite the similar growth fitness of the two resistant strains on cellobiose, more genes specific to L502-6 were regulated than in L502-1 (Figure 3A, Table S1). Together, these results may indicate that the incidence(s) giving rise to the sakacin P resistance in L502-6 resulted in a more complex trait(s) than in L502-1.

### Virulence potential

**Figure 4:**
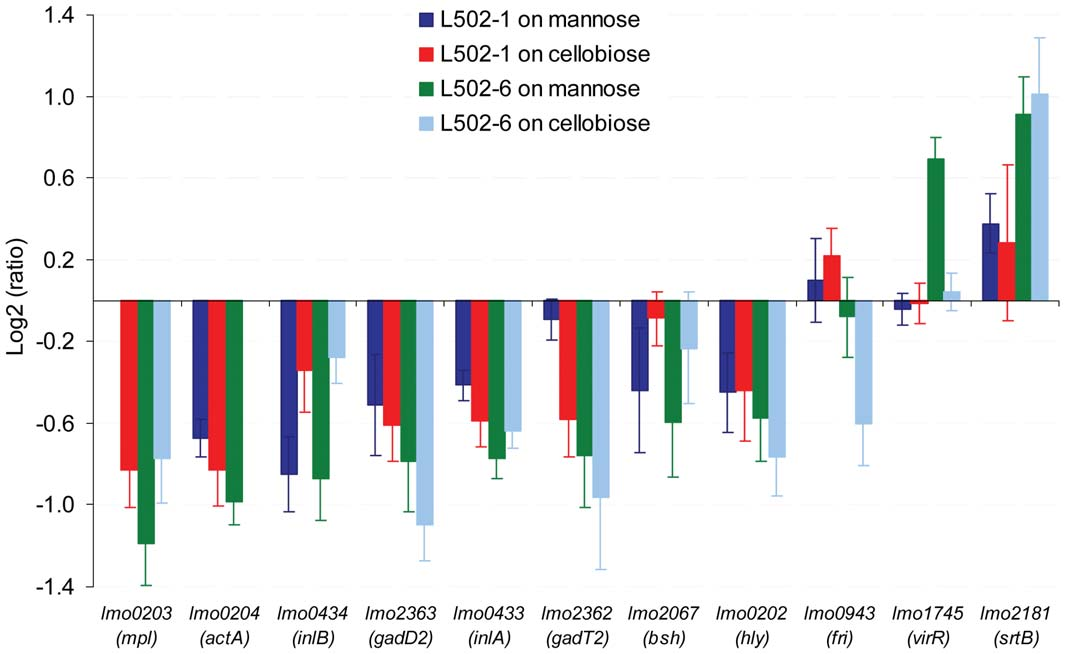
Differentially regulated virulence determinant genes. Virulence determinant genes regulated in the sakacin P-resistant strains in any of the growth conditions compared to the wild-type strain grown on the same sugar. The associated standard deviation (error bars) was computed from the average log ratio and t-value. List of virulent determinate genes were taken from reference [25].

**Figure 5:**
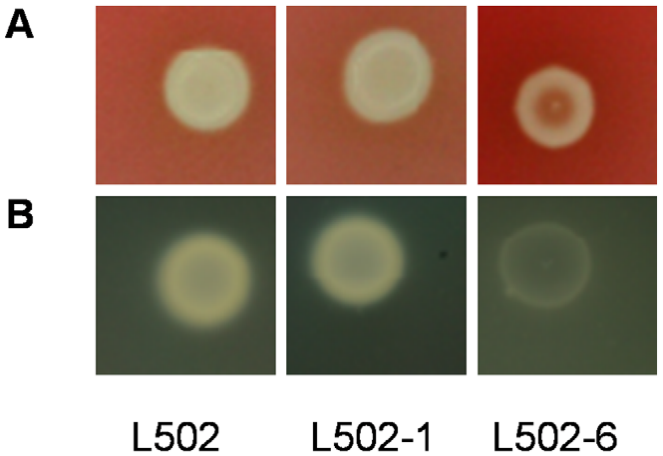
*In vitro* hemolytic and lecithinase activities. (**A**) Hemolytic activity on thin blood agar and (**B**) Lecithinase activity on egg-yolk agar of the wild-type (L502) and the sakacin P-resistant strains (L502-1 and L502-6). Images are a representative of two independent experiments each consisting of duplicates with reproducible results.

In the present study, known and putative virulence determinant genes were significantly down-regulated in the resistant strains compared to that of the wild-type (Figure 4). The proteins encoded by the repressed genes have known roles in the infectious lifecycle of *L. monocytogenes* [25]. For example, the glutamate decarboxylase system (encoded by the *gadT2D2*) and the bile salt hydrolase (*bsh*) could help the bacterium to pass through the alimentary canal and the proteins encoded by *inlA*, *inlB*, *hly*, *actA* and *mpl* could facilitate the adhesion, invasion and cell-to-cell spread of the bacterium [43]. The decreased expression in the *gadT2D2* operon (Figure 4) is partly in line with our earlier finding that showed an increased susceptibility of the sakacin P-resistant strains to low pH stress than the wild-type [20]. No expression data was available for *plcB* gene which encodes phospholipase C, however, the diminished lecithinase activity (Figure 5B) suggested a decrease in expression of the *plcB* gene. In the present study, the expression of *virR* (virulence regulator) and *srtB* genes were up-regulated in L502-6 compared to the wild-type (Figure 4). The VirR and SrtB are associated with modification of the cell surface [45], [46], but the significance of the proteins encoded by these genes in the sakacin P-resistant strain remained to be investigated.

Although qualitative, the results in Figure 5 show that the hemolytic and lecithinase activity of L502-6 were apparently lower than that of the wild-type. In contrast, no difference was observed between L502-1 and the wild-type. The difference between the resistant strains could be attributed to the level of gene transcript (Figure 4) and/or growth fitness (Figure 1, Table 1, [20]). To rule out the latter possibility, an independent hemolytic assay was performed in the cell supernatant after the strains were grown in the presence of cellobiose, which gives a similar growth fitness (Figure 1, Table 1, [20]). In both sakacin P-resistant strains the hemolytic activity was slightly reduced compared to the wild-type, albeit statistically insignificant (p = 0.3) (not shown). A recent study [21], [22] has shown no change in the hemolytic activity after subjecting a *L. monocytogenes* strain to the class IIa bacteriocin sakacin 1. Overall, it is possible that the reduced growth capacity of L502-6 might contribute to the reduced virulence. Further work on the virulence potential of the sakacin P-resistant strains using animal models will be highly informative.

In the present study, the addition of activated charcoal had no apparent effect on hemolytic and lecithinase activities of the strains (not shown). Surprisingly, activated charcoal inhibited the growth of L502-6 by approximately 95% compared to the wild-type (not shown). Removal of important culture components by activated charcoal and/or physical adsorption of the low level sakacin P-resistant strain to the activated charcoal could be the reasons. Thus, in contrast to the previous transcriptional study on class IIa bacteriocin-resistant mutants [19], the present transcriptome study was performed in the absence of activated charcoal. Since activated charcoal may affect the growth fitness of sakacin P-resistant strains, care has to be exercised during evaluation of the virulence potential of such strain, particularly in the presence of activated charcoal.

### Infection with the virulent phage P100

**Figure 6:**
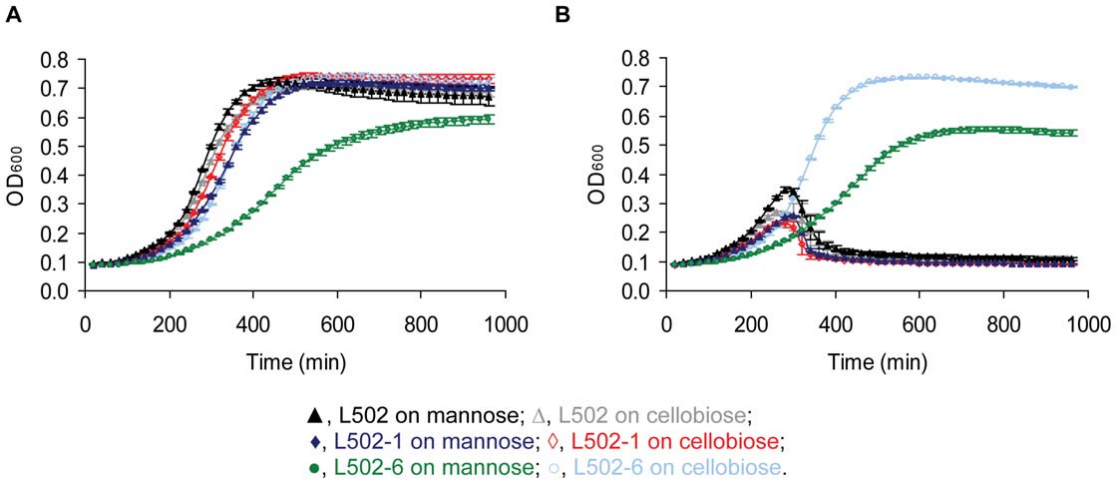
Infection with the lytic bacteriophage P100. Growth of the sakacin P-resistant strains and the wild-type (**A**) not infected with the phage P100 and (**B**) infected with phage P100 at MOI of 10^−9^. Values shown are mean and standard error of the mean (error bars) of a representative of two independent experiments with six measurements each.

Bacteriophage P100 is a strict virulent phage of *L. monocytogenes* and infection inevitably leads to cell death [47]. We assessed if the resistance to sakacin P can cross-protect *L. monocytogenes* from phage infection using Listex P100, a commercial phage approved by the United States Food and Drug Administration as a food biopreservative for the control of *L. monocytogenes* [48]. At higher MOI (multiplicity of infection) all the strains were killed by the phage P100 (not shown). To our surprise, L502-6 showed reproducibly reduced susceptibility to low doses of phage P100 in contrast to the other strains. The killing effect of phage P100 was not dependent on the growth sugar (Figure 6). A similar leaky resistance type to lambda phage has been observed in *Escherichia coli* lacking the membrane components of the mannose PTS [49]. To obtain a complete understanding about the mechanism(s) that underline the reduced susceptibility in L502-6 to low doses of phage P100 requires further investigation.

### Stability of the sakacin P resistance phenotype

During growth in the absence of selective pressure (sakacin P), the sakacin P resistant phenotype in L502-1 started to revert after approximately 20 generations. In contrast, the sakacin P resistance in L502-6 was stable up to 100 generations (not shown). This indicates that the low level resistant strain had a more stable sakacin P resistance phenotype than the high level resistant strain. This contradicts our previous observation which showed more stable phenotype of the high level sakacin P-resistant strains of *L. monocytogenes* L40 and EGDe than the low level resistant strains [20]. As described above, the low level sakacin P-resistant strain studied in the present study (L502-6) may have additional sakacin P-resistance mechanisms, indicating a possible factor that determines the phenotype stability.

The sakacin-P resistant strains are generated after the wild-type strains were exposed to a cell free supernatant (CFS) containing sakacin P prepared by heterologous expression of sakacin P in *Lactobacillus sakei* Lb790(pMLS114) [20]. In the present study, CFS from the non-bacteriocinogenic *L. sakei* Lb790(pLPV111) was used as a negative control [50] and no antilisterial effect was observed in the presence of the CFS from the non-sakacin P producer strain culture (not shown).

### Concluding remarks

The present study reports the first genome-wide transcriptome changes in two different spontaneous sakacin P-resistant mutant strains of *L. monocytogenes*. The experiment was done in the presence of mannose or cellobiose and this facilitated systematic identification of genes possibly related to the bacteriocin resistance. Our data suggested that, for both resistant strains, absence of a normal functional *mpt* as the major mechanism for the sakacin P resistance. In addition, modification of the cell envelope and efflux of the bacteriocin by TAT system might also contribute to the resistance phenotype in the low level sakacin P-resistant strain. The transcriptomics suggested a possible role of the mannose-PTS in global carbon catabolite control in *L. monocytogenes*.

The high level and the low level sakacin P-resistant strains show substantial difference in gene expression profile, stability of the sakacin P resistance phenotype, resistance to phage P100 infection, virulence potential, and growth in the presence of activated charcoal. In our previous study [20], it was shown that the resistant strains displayed different level of resistance to sakacin P, stress tolerance capacity, biofilm formation ability, and Fourier transform infrared spectroscopy profile. Overall, this indicates that the incidence(s) giving rise to the sakacin P resistance involves a complex regulatory gene network and have pleiotropic effects on the physiology of the resistant strains. It would be of interest to extend this study to link the resistance phenotype with mutation(s) in the DNA sequence. Further works on the virulence potential of spontaneous class IIa bacteriocin-resistant strains using animal models would have a practical importance.

## Materials and Methods

### Bacterial strains and growth conditions

The wild-type *L. monocytogenes* strain L502, a cheese isolate of serotype 1/2a and its two spontaneous sakacin P-resistant mutants have been described previously [20], [51]. The mutants were derived after exposure to the class IIa bacteriocin sakacin P [20], and relevant characteristics are summarized in Table 1. The sakacin P producer *L. sakei* Lb790(pMLS114) and its isogenic non-sakacin P producer *L. sakei* Lb790(pLPV111) [50] were used to produce the CFS.

Growth media as well as growth and storage conditions were as previously described [20]. Briefly, *L. monocytogenes* strains were cultivated aerobically at 30°C and liquid cultures were shaken at 200 r.p.m., unless stated otherwise. For hemolytic and lecithinase activity testing, culture media were modified by adding activated charcoal to a final concentration of 0.2% (wt/vol) (Sigma-Aldrich, Amersfoort, The Netherlands) to the Luria-Bertani (LB) broth supplemented with cellobiose. Trypticase soy agar (TSA) was modified by adding either 5% defibrinated horse blood (TCS Biosciences Ltd, Buckingham, UK), 10% egg yolk or 30% CFS from a *L. sakei* culture. The production of CFS from *L. sakei* was done as previously described [20].

### Sampling and RNA isolation

The transcriptome study was performed on *L. monocytogenes* strains grown in LB broth supplemented with mannose or cellobiose (0.5%, wt/vol) in the absence of bacteriocin. Sampling was done at mid-exponential phase (Figure 1) and total RNA extraction was done using an RNeasy Protect bacterial mini kit (Qiagen; [20]). The sakacin P-resistance phenotype was verified on selective plates containing sakacin P at the time of harvesting for RNA isolation. The quantity and quality of RNA was checked using NanoDrop ND-1000 (NanoDrop Technologies, Rockland, USA) and Agilent 2100 Bioanalyzer (Agilent Technologies, Santa Clara, USA). The isolated RNA sample was used both for the microarray and for validation assays by qRT-PCR. Two independently isolated RNA samples from each growth condition were used.

### Microarray analysis

The PCR based DNA microarray slides were developed and printed by The Bacterial Microarray Group at St George's (BμG@S), University of London, UK. Protocols for the development and printing of the microarray slides have been described elsewhere [52], [53]. The array design is available in BμG@Sbase (Accession No. A-BUGS-19; http://bugs.sgul.ac.uk/A-BUGS-19) and also ArrayExpress (Accession No. A-BUGS-19). The cDNA synthesis and labeling were performed using 4 µg total RNA following established method [53]. The sakacin P-resistant mutants grown on mannose or cellobiose were compared to the wild-type strain grown on the respective sugar. Dye-swap hybridization was performed for each sample and the hybridization was done as described previously [52], [53]. Scanning of the arrays was performed using Tecan LS scanner (Tecan, Männedorf, Switzerland). Spot-identification, -segmentation and -fluorescent intensity quantification were done using ImaGene 5.5 (BioDiversity, El Segundo, USA).

The microarray data analysis was done by the LIMMA package [54] in the R computing environment (http://www.r-project.org/). Preprocessing and normalization followed a standard procedure using methods described by Smyth and Speed [55]. Testing for differentially expressed genes was done using a linear mixed model as described [56]. A mixed-model approach was chosen to adequately describe between-array variation, and utilized the probe-replicates in each array. An empirical Bayes smoothing of gene-wise variances was conducted according to Smyth et al. [57]. For each gene, the p-value was adjusted to control the false discovery rate and the adjusted p-values were referred as q-values. Differentially expressed genes were selected using q-values less than 0.01 and with log ratio of ≥0.585 or ≤−0.585 (equivalent to ≥1.5-fold changes).

The preprocessed data were also analyzed using The Unscrambler software (The Unscrambler v9.8; CAMO AS, Norway), GeneSpring GX7.3 (Agilent Technologies, Santa Clara, USA) and the LEGER database [58]. For the PCA, genes that were differentially expressed in any of the growth conditions were included. Hierarchical clustering (complete linkage, correlation distance metric) of all differentially expressed genes was used to generate a heatmap. Genes were grouped according to the role category annotation in CMR-JCVI *L. monocytogenes* EGDe genome database (http://cmr.jcvi.org). Fisher's exact test and odd ratios analysis was performed to determine significantly enriched gene compared to the CMR-JCVI role category. Known and putative genes involved in virulence were identified from the recent genome-wide transcriptional studies [25].

### Hemolytic activity assay

An agar based hemolytic assay was performed on the overnight cultures grown in the LB broth supplemented with cellobiose. The blood agar was divided into four sections, and equal volume of bacterial suspensions of the wild-type and the sakacin P-resistant strains were spotted onto each quadrant. A no-growth control (only the broth) and parallel inoculation of the bacterial suspensions onto TSA were included as a control. All the plates were incubated at 37°C and halos due to hemolytic activity were checked visually after 16–18 h. The assay was done twice on different days in duplicate. Hemolytic activity in the supernatant from the overnight cultures grown in the LB broth supplemented with cellobiose and activated charcoal was analyzed as described previously [38]. A total of six measurements were performed on two samples collected on different days. Statistical significance test was performed using a t-test. Viable cell count was performed to check if the addition of activated charcoal had effect on growth.

### Lecithinase activity assay

A lecithinase activity assay was performed on agar containing egg-yolk [59]. The TSA egg-yolk agars were inoculated with the bacterial culture suspension following the same protocol described for the agar based hemolytic activity assay. Halos due to lecithinase activity were estimated after 72 h of incubation at 37°C. The assay was done in two biological duplicates each consisting at least two technical duplicates.

### Infection with Listex P100

The *L. monocytogenes* strains were infected with bacteriophage P100 in LB broth supplemented with mannose or cellobiose. Phage titration of the stock solution of Listex P100 was performed following the instruction provided by the supplier (EBI food safety, Wageningen, The Netherlands). The *L. monocytogenes* strains were prepared as described earlier [20] and were infected with 10, 10^−1^, 10^−6^ and 10^−9^ MOI. Growth was monitored using a Bioscreen C instrument (Oy Growth Curves Ab Ltd., Helsinki, Finland) at 30°C for 18 h as described [20]. The assay was done twice on different days each consisting six replicates.

### Stability of sakacin P resistance

The stability of the sakacin P resistance phenotype of L502-1 and L502-6 was performed as described before [20] with a slight modification. In the present study, CFS from the non-bacteriocinogenic *L. sakei* Lb790(pLPV111) used as a negative control [50]. The stability assay was performed in triplicates on different days.

### Validation of microarray data by qRT-PCR assay

The microarray results were validated on six selected genes by qRT-PCR performed as previously described [20]. Selection of genes for the validation assay was following the guidelines suggested by Morey et al. [60]. The primer and probe sets for *actA* (encoding actin assembly-inducing protein), *lmo1251* (encoding a putative CRP-FNR family transcriptional regulator), *glpK* (glycerol kinase) and *kdpA* (potassium-transporting ATPase A chain) were designed using Primer Express 3.0. The sets for the *mptA* and 16S rRNA have been reported earlier [20] (Table S2).

### Microarray data accession number

Microarray data are MIAME compliant. Fully annotated microarray data have been deposited in BμG@Sbase (accession number E-BUGS-110; http://bugs.sgul.ac.uk/E-BUGS-110) and also ArrayExpress (accession number E-BUGS-110).

## Supporting Information

Figure S1 Total number of regulated genes grouped according to their biological role. Total number of (**A**) up-regulated and (**B**) down-regulated genes in different functional role categories according to the primary annotation in CMR-JCVI *L. monocytogenes* EGDe genome database in the L502-1 and L502-6 strains grown on mannose or cellobiose compared to the wild-type grown on the respective sugar. A, Amino acid biosynthesis; B, Biosynthesis of cofactors, prosthetic groups, and carriers; C, Cell envelope; D, Cellular processes; E, Central intermediary metabolism; H, Energy metabolism; I, Fatty acid and phospholipid metabolism; J, Hypothetical proteins; L, Mobile and extrachromosomal element functions; M, Protein fate; N, Protein synthesis; O, Purines, pyrimidines, nucleosides, and nucleotides; P, Regulatory functions; Q, Signal transduction; S, Transport and binding proteins; T, Unclassified; U, Unknown function; V, Viral functions.
(TIF)

Figure S2 Validation of the microarray data by qRT-PCR analysis. Validation of microarray data (y-axis) by qRT-PCR analysis (x-axis) on six genes. The transcriptional studies for the sakacin P-resistant strains (L502-1 and L502-6) and the wild-type (L502) was performed during growth on mannose or cellobiose. (**A**) L502-1 grown on mannose (**B**) L502-1 grown on cellobiose, (**C**) L502-6 grown on mannose, and (**D**) L502-6 grown on cellobiose and all the log2 ratio values are relative to the wild-type (L502) grown on the respective sugars. The data presented here represent results from two biological replicates.
(TIF)

Figure S3 Transcript level of the *mptA* gene. Transcript level of the *mptA* gene extracted from qRT-PCR analysis in the sakacin P-resistant (L502-1 and L502-6) and the wild-type (L502) strain during growth on mannose or cellobiose. Error bar indicates the standard error of the mean of two independent experiments.
(TIF)

Table S1 List of all differentially regulated genes. ^a^ Description of genes according to the annotation of the Comprehensive Microbial Resource of the J. Craig Venter Institute (http://cmr.jcvi.org) and published literature resources. The functional role categories are also according to the primary annotation in CMR-JCVI *L. monocytogenes* EGDe genome database. A, Amino acid biosynthesis; B, Biosynthesis of cofactors, prosthetic groups, and carriers; E, Central intermediary metabolism; H, Energy metabolism; I, Fatty acid and phospholipid metabolism; J, Hypothetical proteins; N, Protein synthesis; O, Purines, pyrimidines, nucleosides, and nucleotides; Q, Signal transduction. ^b^ Log2 expression ratio [a high level sakacin P-resistant strain (L502-1) and a low level sakacin P-resistant strains (L502-6) grown on mannose (M) or cellobiose (C) relative to the wild-type (L502) grown on the respective sugars]; values in bold face indicate differentially expressed genes as defined as log2 ratios ≥0.585 or ≤−0.585 and with q-value<0.01, p-value adjusted to control false discovery rate). NA, no data available. Fully annotated microarray data have been deposited in BμG@Sbase (accession number E-BUGS-110; http://bugs.sgul.ac.uk/E-BUGS-110) and also ArrayExpress (accession number E-BUGS-110).
(DOC)

Table S2 Primer and probe sets used for qRT-PCR. ^a^ Taq probes, 6-FAM, 6-carboxyfluorescein (fluorophore); TAMRA, 6-carboxytetramethylrhodamine (quencher).
(DOC)
