# Supplementary figures and images for: Global Transcriptional Analysis of Spontaneous Sakacin P-Resistant Mutant Strains of Listeria monocytogenes during Growth on Different Sugars
Source: PLoS One. 2011 Jan 6;6(1):e16192. doi: 10.1371/journal.pone.0016192 (PMC3017082; doi:10.1371/journal.pone.0016192)

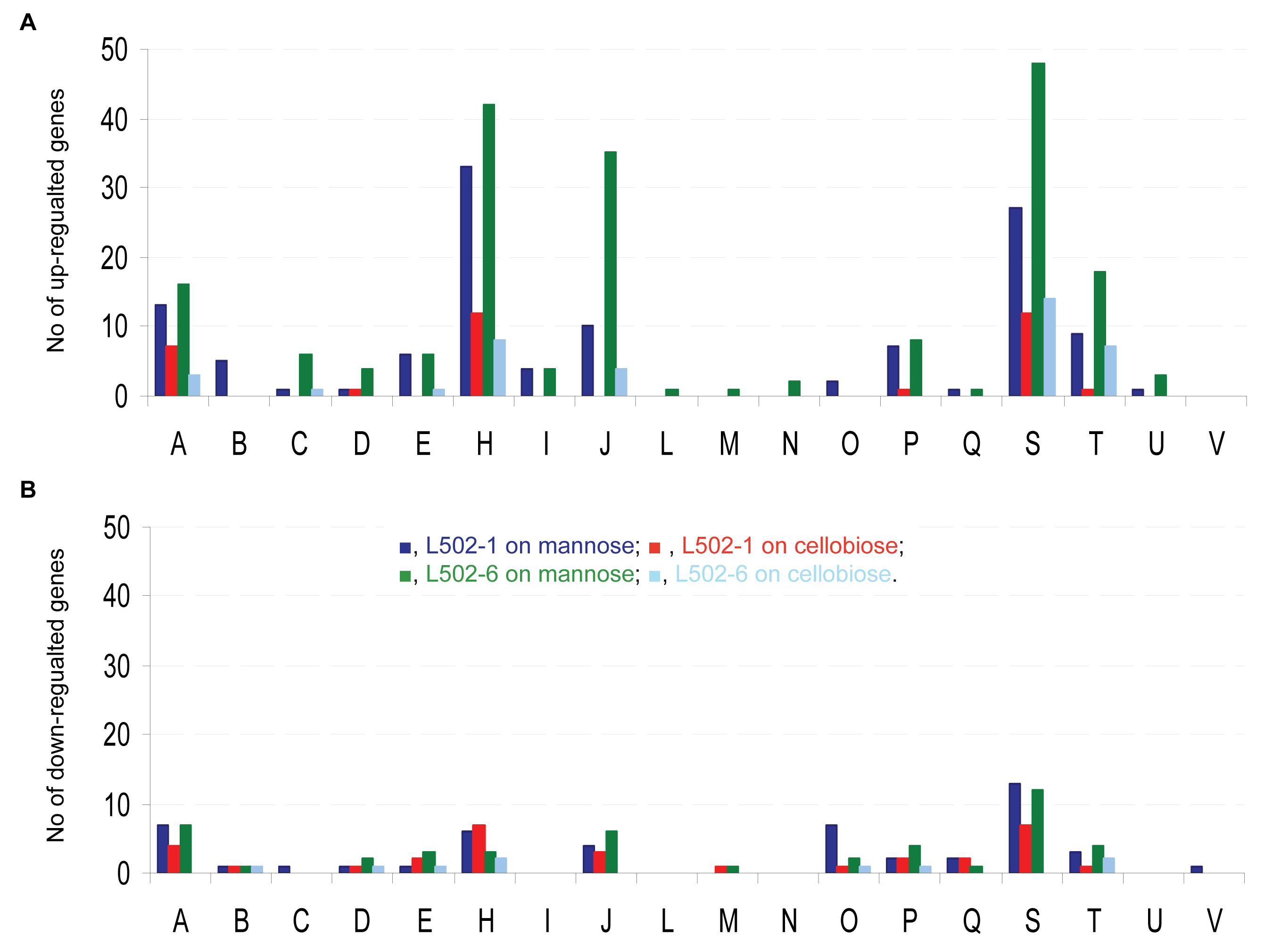

Supplement: Figure S1 — Total number of regulated genes grouped according to their biological role. Total number of (A) up-regulated and (B) down-regulated genes in different functional role categories according to the primary annotation in CMR-JCVI L. monocytogenes EGDe genome database in the L502-1 and L502-6 strains grown on mannose or cellobiose compared to the wild-type grown on the respective sugar. A, Amino acid biosynthesis; B, Biosynthesis of cofactors, prosthetic groups, and carriers; C, Cell envelope; D, Cellular processes; E, Central intermediary metabolism; H, Energy metabolism; I, Fatty acid and phospholipid metabolism; J, Hypothetical proteins; L, Mobile and extrachromosomal element functions; M, Protein fate; N, Protein synthesis; O, Purines, pyrimidines, nucleosides, and nucleotides; P, Regulatory functions; Q, Signal transduction; S, Transport and binding proteins; T, Unclassified; U, Unknown function; V, Viral functions. (TIF) [file pone.0016192.s001.tif]

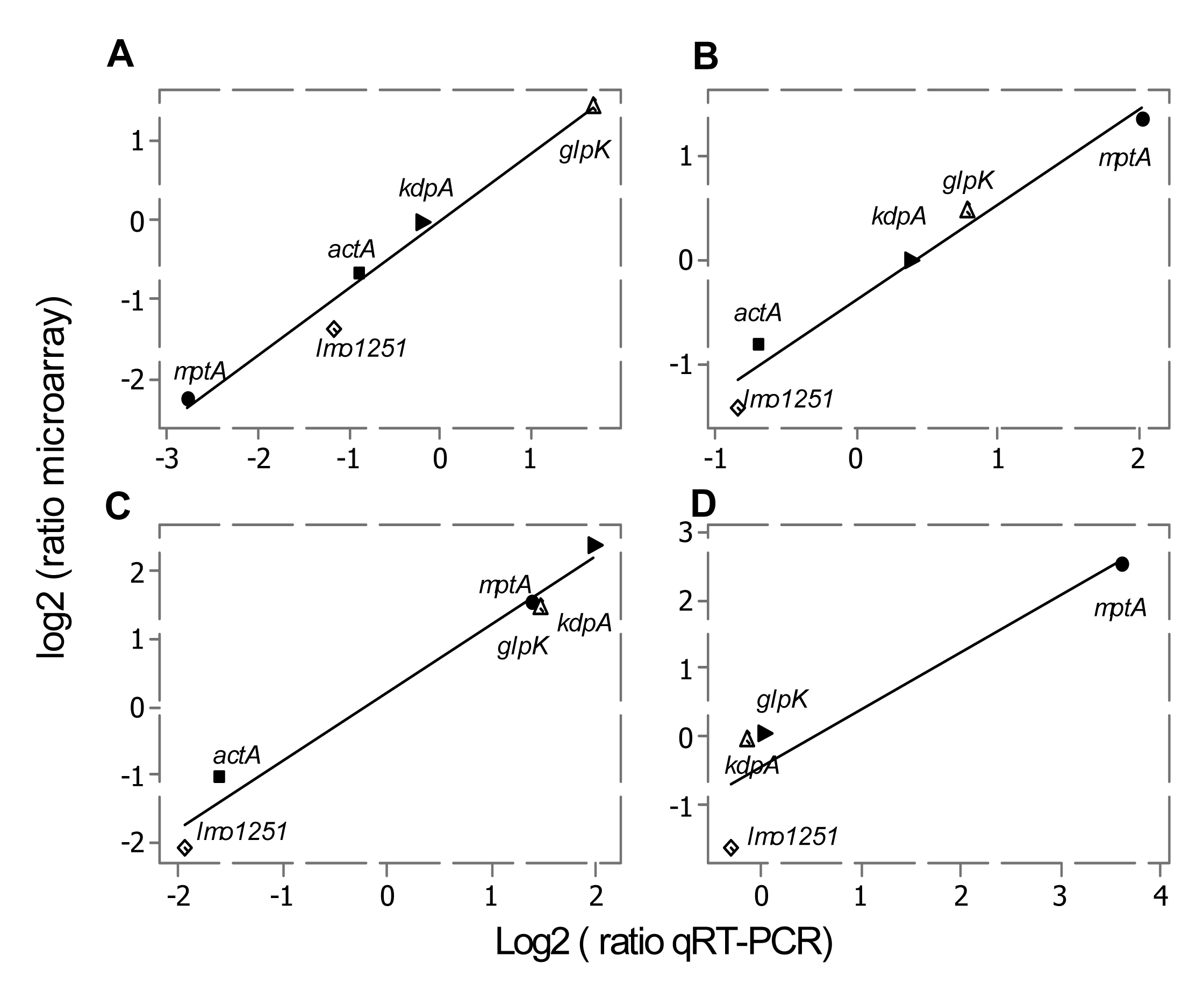

Supplement: Figure S2 — Validation of the microarray data by qRT-PCR analysis. Validation of microarray data (y-axis) by qRT-PCR analysis (x-axis) on six genes. The transcriptional studies for the sakacin P-resistant strains (L502-1 and L502-6) and the wild-type (L502) was performed during growth on mannose or cellobiose. (A) L502-1 grown on mannose (B) L502-1 grown on cellobiose, (C) L502-6 grown on mannose, and (D) L502-6 grown on cellobiose and all the log2 ratio values are relative to the wild-type (L502) grown on the respective sugars. The data presented here represent results from two biological replicates. (TIF) [file pone.0016192.s002.tif]

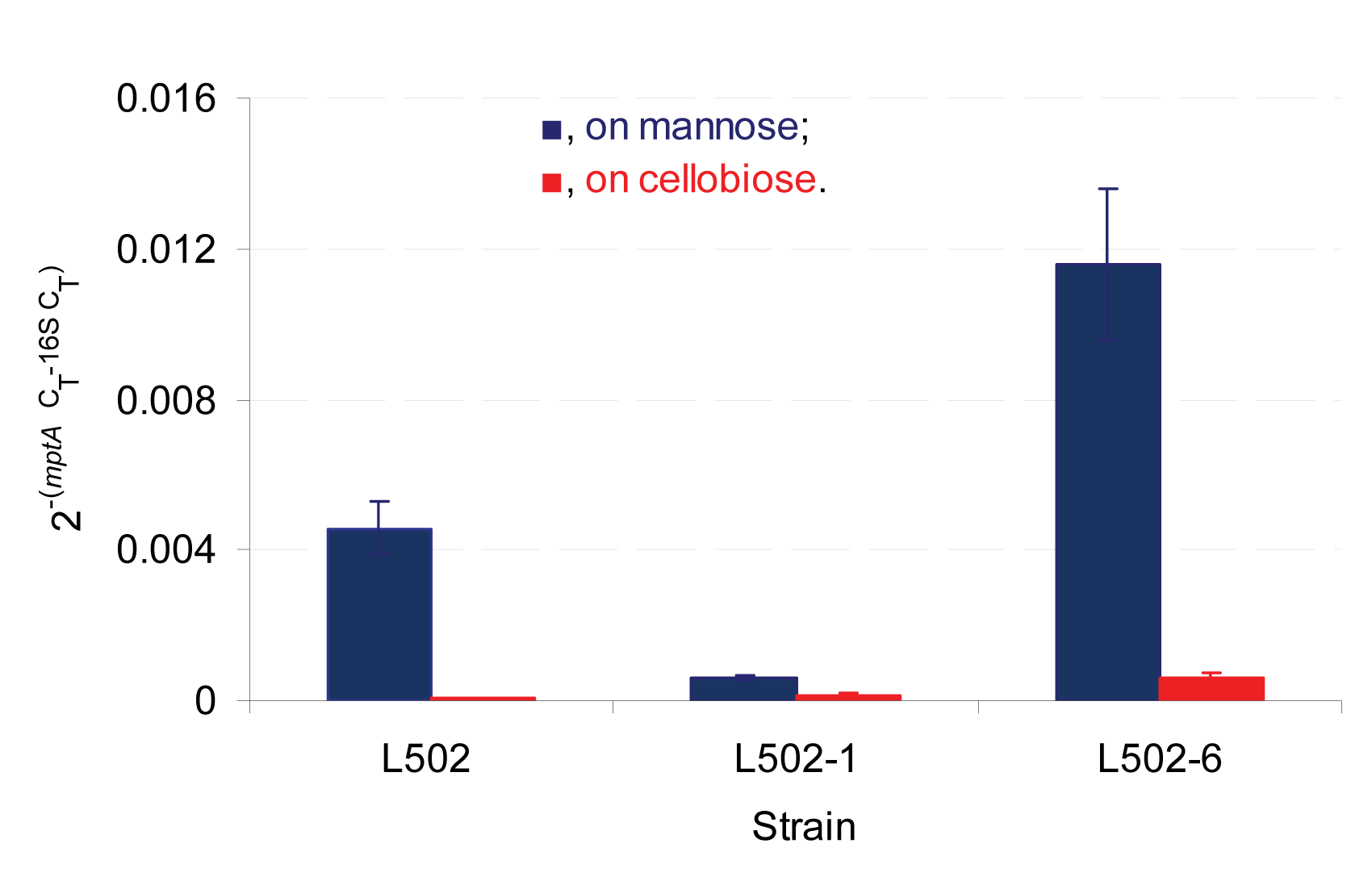

Supplement: Figure S3 — Transcript level of the mptA gene. Transcript level of the mptA gene extracted from qRT-PCR analysis in the sakacin P-resistant (L502-1 and L502-6) and the wild-type (L502) strain during growth on mannose or cellobiose. Error bar indicates the standard error of the mean of two independent experiments. (TIF) [file pone.0016192.s003.tif]
