# Supplementary material for: Global Transcriptional Analysis of Spontaneous Sakacin P-Resistant Mutant Strains of Listeria monocytogenes during Growth on Different Sugars
Source: PLoS One. 2011 Jan 6;6(1):e16192. doi: 10.1371/journal.pone.0016192 (PMC3017082; doi:10.1371/journal.pone.0016192)
Supplement: Table S1 — List of all differentially regulated genes. a Description of genes according to the annotation of the Comprehensive Microbial Resource of the J. Craig Venter Institute (http://cmr.jcvi.org) and published literature resources. The functional role categories are also according to the primary annotation in CMR-JCVI L. monocytogenes EGDe genome database. A, Amino acid biosynthesis; B, Biosynthesis of cofactors, prosthetic groups, and carriers; E, Central intermediary metabolism; H, Energy metabolism; I, Fatty acid and phospholipid metabolism; J, Hypothetical proteins; N, Protein synthesis; O, Purines, pyrimidines, nucleosides, and nucleotides; Q, Signal transduction. b Log2 expression ratio [a high level sakacin P-resistant strain (L502-1) and a low level sakacin P-resistant strains (L502-6) grown on mannose (M) or cellobiose (C) relative to the wild-type (L502) grown on the respective sugars]; values in bold face indicate differentially expressed genes as defined as log2 ratios ≥0.585 or ≤−0.585 and with q-value<0.01, p-value adjusted to control false discovery rate). NA, no data available. Fully annotated microarray data have been deposited in BμG@Sbase (accession number E-BUGS-110; http://bugs.sgul.ac.uk/E-BUGS-110) and also ArrayExpress (accession number E-BUGS-110). (DOC) [file pone.0016192.s004.doc]

|  |  |  |  | **L502-1 (M)^b^** | | **L502-1 (C)** | | **L502-6 (M)** | | **L502-6 (C)** | |
| --- | --- | --- | --- | --- | --- | --- | --- | --- | --- | --- | --- |
| **Locus** | **Gene** | **Description (similar to)^a^** | **Role(s)^a^** | **Log2** | **q-value** | **Log2** | **q-value** | **Log2** | **q-value** | **Log2** | **q-value** |
| **Amino acid biosynthesis** | | | | | | | | | | | |
| lmo1588 | *argD* | N-acetylornithine aminotransferase |  | -0.45 | 0.028 | -0.26 | 0.046 | **-0.92** | **0.001** | -0.11 | 0.462 |
| lmo1589 | *argB* | N-acetylglutamate 5-phosphotransferase |  | -0.35 | 0.167 | -0.21 | 0.002 | **-0.98** | **0.001** | 0.02 | 0.862 |
| lmo1590 | *argJ* | ornithine acetyltransferase and amino-acid acetyltransferases |  | NA | NA | NA | NA | **-0.62** | **0.006** | -0.06 | 0.614 |
| lmo2006 | *alsS* | alpha-acetolactate synthase protein, AlsS |  | **-0.93** | **0.000** | -0.32 | 0.001 | **-1.18** | **0.000** | 0.17 | 0.183 |
| lmo2090 | *argG* | argininosuccinate synthase |  | -0.42 | 0.000 | -0.53 | 0.011 | **-1.27** | **0.000** | 0.58 | 0.041 |
| lmo2091 | *argH* | argininosuccinate lyase |  | -0.29 | 0.308 | -0.35 | 0.012 | **-1.35** | **0.001** | 0.18 | 0.422 |
| lmo2252 |  | aspartate aminotransferase |  | **-0.59** | **0.000** | -0.47 | 0.000 | **-1.44** | **0.000** | -0.21 | 0.004 |
| **Biosynthesis of cofactors, prosthetic groups, and carriers** | | | | | | | | | | | |
| lmo1042 |  | molybdopterin biosynthesis protein moeA |  | **0.64** | **0.000** | 0.12 | 0.220 | 0.52 | 0.000 | 0.02 | 0.840 |
| lmo1043 |  | molybdopterin-guanine dinucleotide biosynthesis MobB |  | **0.69** | **0.000** | 0.15 | 0.036 | 0.51 | 0.000 | 0.04 | 0.493 |
| lmo1045 |  | molybdopterin converting factor (subunit 1). |  | **0.59** | **0.000** | 0.11 | 0.152 | 0.39 | 0.000 | 0.00 | 0.971 |
| lmo1046 | *moaC* | molybdenum cofactor biosynthesis protein C |  | **0.60** | **0.000** | 0.14 | 0.066 | 0.50 | 0.000 | 0.04 | 0.568 |
| lmo1047 |  | molybdenum cofactor biosynthesis protein A |  | **0.64** | **0.000** | 0.15 | 0.028 | 0.49 | 0.000 | -0.02 | 0.804 |
| lmo1194 | *cbiD* | cobalamin biosynthesis protein CbiD |  | **-0.68** | **0.001** | -0.39 | 0.054 | 0.06 | 0.233 | -0.02 | 0.832 |
| **Cell envelope** | | | | | | | | | | | |
| lmo0129 |  | autolysin: N-acetylmuramoyl-L-alanine amidase |  | **-0.73** | **0.000** | -0.30 | 0.104 | 0.11 | 0.386 | -0.23 | 0.220 |
| lmo0195 |  | membrane protein (putative ABC transporter) |  | -0.16 | 0.017 | 0.01 | 0.913 | **1.90** | **0.000** | 0.10 | 0.109 |
| lmo0366 |  | conserved hypothetical protein, putative lipoprotein |  | 0.39 | 0.002 | 0.38 | 0.273 | **1.14** | **0.000** | **1.23** | **0.001** |
| lmo0880 |  | wall associated protein precursor (LPXTG motif) |  | 0.12 | 0.155 | 0.06 | 0.883 | **1.02** | **0.001** | -0.03 | 0.826 |
| lmo1044 |  | molybdopterin converting factor, subunit 2 |  | **0.66** | **0.000** | 0.13 | 0.298 | 0.53 | 0.000 | 0.00 | 0.990 |
| lmo1215 |  | N-acetylmuramoyl-L-alanine amidase (autolysin) |  | -0.10 | 0.065 | -0.10 | 0.169 | **1.10** | **0.000** | -0.10 | 0.066 |
| lmo2229 |  | penicillin-binding protein |  | -0.06 | 0.483 | -0.04 | 0.683 | **0.89** | **0.000** | -0.01 | 0.891 |
| lmo2484 |  | *B. subtilis* YvlD protein |  | -0.08 | 0.394 | -0.09 | 0.214 | **2.73** | **0.000** | -0.01 | 0.958 |
| **Cellular processes** | | | | | | | | | | | |
| lmo0028 |  | *E. coli* microcin C7 self-immunity protein (MccF) |  | 0.07 | 0.398 | **2.24** | **0.000** | -0.30 | 0.114 | 0.25 | 0.069 |
| lmo0433 | *inlA* | internalin A |  | -0.41 | 0.000 | **-0.59** | **0.000** | **-0.77** | **0.000** | **-0.64** | **0.000** |
| lmo0434 | *inlB* | internalin B |  | **-0.85** | **0.000** | -0.34 | 0.088 | **-0.87** | **0.001** | -0.27 | 0.035 |
| lmo0601 |  | cell surface protein |  | -0.04 | 0.483 | -0.09 | 0.430 | **0.68** | **0.000** | -0.01 | 0.951 |
| lmo1879 | *cspD* | cold shock protein |  | **1.21** | **0.000** | 0.36 | 0.002 | **0.84** | **0.000** | 0.06 | 0.773 |
| lmo1967 |  | toxic ion resistance proteins |  | -0.10 | 0.271 | -0.06 | 0.556 | **1.99** | **0.000** | 0.20 | 0.397 |
| lmo2230 |  | arsenate reductase |  | -0.01 | 0.928 | 0.06 | 0.622 | **0.90** | **0.000** | 0.04 | 0.784 |
| **Central intermediary metabolism** | | | | | | | | | | | |
| lmo0372 |  | beta-glucosidase |  | **-1.85** | **0.000** | 0.10 | 0.304 | **-2.21** | **0.000** | 0.06 | 0.818 |
| lmo0574 |  | beta-glucosidase |  | 0.25 | 0.001 | **-0.97** | **0.000** | 0.15 | 0.038 | -0.21 | 0.019 |
| lmo0877 |  | *B. subtilis* NagB protein |  | **0.61** | **0.001** | 0.21 | 0.053 | 0.50 | 0.011 | 0.04 | 0.515 |
| lmo0878 |  | oxidoreductases |  | **0.68** | **0.000** | 0.22 | 0.023 | 0.50 | 0.000 | 0.03 | 0.671 |
| lmo2555 |  | human N-acetylglucosaminyl-phosphatidylinositol biosynthetic protein | N | -0.15 | 0.002 | -0.09 | 0.215 | **0.80** | **0.000** | 0.03 | 0.620 |
| lmo2721 |  | glucosamine-6-phosphate isomerase |  | -0.50 | 0.001 | -0.25 | 0.001 | **-0.62** | **0.000** | -0.27 | 0.091 |
| lmo2798 |  | phosphatase |  | **0.84** | **0.000** | 0.17 | 0.160 | **0.87** | **0.001** | 0.28 | 0.056 |
| **Energy metabolism** | | | | | | | | | | | |
| lmo0105 |  | chitinase B |  | 0.56 | 0.033 | **-1.61** | **0.000** | **1.02** | **0.000** | -0.09 | 0.166 |
| lmo0261 |  | phospho-beta-glucosidase |  | 0.53 | 0.000 | 0.14 | 0.059 | **0.63** | **0.000** | 0.19 | 0.018 |
| lmo0319 |  | phospho-beta-glucosidase |  | 0.31 | 0.000 | **2.61** | **0.000** | 0.25 | 0.010 | 0.49 | 0.014 |
| lmo0343 |  | transaldolase |  | **0.84** | **0.000** | 0.32 | 0.003 | **0.81** | **0.000** | 0.25 | 0.006 |
| lmo0344 |  | dehydrogenase/reductase |  | **0.80** | **0.000** | 0.36 | 0.005 | **0.74** | **0.000** | 0.30 | 0.002 |
| lmo0345 |  | sugar-phosphate isomerase |  | **0.96** | **0.000** | 0.27 | 0.006 | **0.75** | **0.000** | 0.11 | 0.686 |
| lmo0346 |  | triosephosphate isomerase |  | 0.60 | 0.013 | 0.37 | 0.090 | **0.63** | **0.004** | 0.14 | 0.342 |
| lmo0347 |  | dihydroxyacetone kinase | I | **0.81** | **0.001** | 0.29 | 0.032 | **0.77** | **0.007** | 0.19 | 0.140 |
| lmo0485 |  |  | E | 0.06 | 0.704 | 0.25 | 0.208 | **0.65** | **0.007** | **0.79** | **0.002** |
| lmo0517 |  | phosphoglycerate mutase |  | 0.25 | 0.097 | **2.45** | **0.000** | NA | NA | 0.79 | 0.010 |
| lmo0643 |  | putative transaldolase |  | **0.78** | **0.000** | 0.37 | 0.006 | 0.42 | 0.000 | 0.04 | 0.832 |
| lmo0813 |  | fructokinases |  | **1.10** | **0.000** | -0.20 | 0.238 | **0.83** | **0.000** | 0.29 | 0.013 |
| lmo0917 |  | beta-glucosidase |  | **0.89** | **0.000** | 0.05 | 0.391 | **1.01** | **0.000** | 0.16 | 0.039 |
| lmo0943 | *fri* | non-heme iron-binding ferritin |  | 0.10 | 0.496 | 0.22 | 0.067 | -0.08 | 0.535 | **-0.60** | **0.008** |
| lmo1254 |  | alpha,alpha-phosphotrehalase |  | **2.30** | **0.000** | **0.59** | **0.000** | **2.01** | **0.000** | 0.08 | 0.224 |
| lmo1293 | *glpD* | Glycerol-3-phosphate dehydrogenase |  | **1.98** | **0.000** | 0.54 | 0.000 | **2.11** | **0.000** | 0.09 | 0.178 |
| lmo1406 | *pflB* | pyruvate formate-lyase |  | -0.20 | 0.091 | **-0.64** | **0.000** | -0.15 | 0.018 | 0.10 | 0.092 |
| lmo1538 | *glpK* | glycerol kinase |  | **1.43** | **0.000** | 0.46 | 0.000 | **1.46** | **0.000** | -0.02 | 0.803 |
| lmo1566 | *citC* | isocitrate dehyrogenases |  | 0.46 | 0.154 | 0.45 | 0.011 | **0.68** | **0.006** | **0.71** | **0.005** |
| lmo1567 | *citZ* | citrate synthase subunit II |  | 0.55 | 0.037 | 0.40 | 0.000 | **0.75** | **0.000** | **0.82** | **0.001** |
| lmo1579 |  | alanine dehydrogenase |  | -0.04 | 0.440 | -0.14 | 0.226 | **0.80** | **0.009** | -0.33 | 0.309 |
| lmo1641 | *citB* | aconitate hydratases |  | **0.59** | **0.001** | 0.47 | 0.003 | **0.84** | **0.000** | **0.85** | **0.000** |
| lmo1992 | *alsD* | alpha-acetolactate decarboxylase |  | -0.43 | 0.000 | -0.24 | 0.001 | **-0.70** | **0.000** | -0.07 | 0.506 |
| lmo2122 |  | maltodextrose utilization protein MalA | A | **0.61** | **0.000** | 0.23 | 0.121 | 0.17 | 0.219 | 0.33 | 0.100 |
| lmo2124 |  | maltodextrin ABC-transport system (permease) |  | **0.68** | **0.000** | 0.20 | 0.207 | 0.34 | 0.001 | 0.35 | 0.038 |
| lmo2159 |  | oxidoreductase | E | **1.11** | **0.000** | 0.25 | 0.027 | **0.82** | **0.000** | 0.34 | 0.020 |
| lmo2163 |  | oxidoreductase | E | **1.28** | **0.000** | 0.34 | 0.058 | **1.07** | **0.000** | 0.41 | 0.035 |
| lmo2363 | *gadD2* | glutamate decarboxylase | B,E,O | -0.51 | 0.021 | **-0.61** | **0.006** | **-0.78** | **0.008** | **-1.10** | **0.001** |
| lmo2436 |  | transcription antiterminator |  | 0.32 | 0.000 | **2.15** | **0.000** | 0.18 | 0.014 | 0.44 | 0.047 |
| lmo2584 |  | formate dehydrogenase associated protein |  | **0.65** | **0.000** | 0.19 | 0.061 | 0.41 | 0.000 | -0.11 | 0.154 |
| lmo2586 |  | formate dehydrogenase alpha chain |  | **1.13** | **0.000** | 0.25 | 0.018 | **0.87** | **0.000** | -0.11 | 0.128 |
| lmo2664 |  | sorbitol dehydrogenase | E | **0.60** | **0.000** | 0.15 | 0.015 | 0.14 | 0.033 | -0.17 | 0.029 |
| lmo2695 |  | dihydroxyacetone kinase | I | **0.61** | **0.000** | 0.16 | 0.070 | **0.95** | **0.000** | 0.05 | 0.370 |
| lmo2696 |  | hypothetical dihydroxyacetone kinase | I | 0.57 | 0.000 | 0.15 | 0.027 | **0.94** | **0.000** | 0.11 | 0.103 |
| lmo2720 | *acs* | acetate-CoA ligase |  | **-0.66** | **0.000** | -0.36 | 0.004 | **-1.02** | **0.000** | -0.21 | 0.011 |
| lmo2743 |  | transaldolase |  | **0.75** | **0.000** | 0.09 | 0.447 | **0.81** | **0.000** | 0.15 | 0.061 |
| lmo2771 |  | beta-glucosidase |  | 0.35 | 0.000 | **0.86** | **0.000** | 0.09 | 0.180 | 0.13 | 0.279 |
| lmo2836 |  | alcohol dehydrogenase | E | 0.29 | 0.001 | 0.16 | 0.339 | **0.65** | **0.002** | 0.23 | 0.440 |
| lmo2840 |  | sucrose phosphorylase | O | **0.64** | **0.001** | 0.26 | 0.057 | 0.47 | 0.000 | 0.34 | 0.028 |

|  |  |  |  | **L502-1 (M)^b^** | | **L502-1 (C)** | | **L502-6 (M)** | | **L502-6 (C)** | |
| --- | --- | --- | --- | --- | --- | --- | --- | --- | --- | --- | --- |
| **Locus** | **Gene** | **Description (similar to)^a^** | **Role(s)^a^** | **Log2** | **q-value** | **Log2** | **q-value** | **Log2** | **q-value** | **Log2** | **q-value** |
| **Fatty acid and phospholipid metabolism** | | | | | | | | | | | |
| lmo0110 |  | lipase |  | **0.90** | **0.001** | 0.36 | 0.041 | **0.74** | **0.005** | 0.06 | 0.858 |
| lmo2175 |  | dehydrogenase |  | **0.60** | **0.001** | 0.39 | 0.004 | 0.45 | 0.030 | -0.14 | 0.354 |
| **Hypothetical proteins** | | | | | | | | | | | |
| lmo0099 |  |  |  | **-1.96** | **0.000** | 0.30 | 0.002 | **1.49** | **0.000** | **0.80** | **0.001** |
| lmo0119 |  |  |  | **-0.70** | **0.000** | -0.23 | 0.104 | -0.20 | 0.478 | NA | NA |
| lmo0120 |  |  |  | -0.97 | 0.012 | **-0.69** | **0.004** | 0.09 | 0.442 | -0.27 | 0.309 |
| lmo0125 |  |  |  | **-0.63** | **0.004** | -0.23 | 0.324 | -0.24 | 0.160 | -0.12 | 0.677 |
| lmo0127 |  | protein gp20 from Bacteriophage A118 |  | **-0.82** | **0.000** | -0.17 | 0.491 | -0.12 | 0.558 | -0.09 | 0.748 |
| lmo0193 |  |  |  | -0.14 | 0.004 | 0.00 | 0.972 | **2.21** | **0.000** | 0.18 | 0.082 |
| lmo0323 |  | unknown proteins |  | **0.64** | **0.000** | 0.41 | 0.000 | **0.77** | **0.001** | 0.25 | 0.046 |
| lmo0335 |  |  |  | NA | NA | NA | NA | **-0.65** | **0.007** | NA | NA |
| lmo0349 |  |  |  | **0.66** | **0.004** | 0.29 | 0.097 | **1.00** | **0.000** | 0.34 | 0.368 |
| lmo0350 |  |  |  | 0.43 | 0.022 | 0.20 | 0.107 | **0.59** | **0.000** | 0.20 | 0.163 |
| lmo0365 |  | conserved hypothetical protein |  | 0.41 | 0.009 | 0.46 | 0.212 | **1.38** | **0.000** | **1.52** | **0.000** |
| lmo0391 |  |  |  | 0.50 | 0.000 | 0.16 | 0.018 | **0.67** | **0.000** | 0.07 | 0.354 |
| lmo0397 |  | unknown proteins |  | 0.48 | 0.000 | -0.07 | 0.305 | **0.70** | **0.000** | 0.07 | 0.309 |
| lmo0412 |  |  |  | -0.64 | 0.025 | -0.39 | 0.002 | **-0.94** | **0.000** | -0.08 | 0.719 |
| lmo0477 |  | putative secreted protein |  | 0.45 | 0.000 | 0.18 | 0.009 | **0.60** | **0.000** | -0.05 | 0.508 |
| lmo0546 |  | putative NAD(P)-dependent oxidoreductase |  | **0.78** | **0.003** | 0.19 | 0.285 | 0.41 | 0.140 | 0.44 | 0.091 |
| lmo0599 |  | conserved hypothetical protein |  | -0.18 | 0.005 | -0.14 | 0.054 | **0.77** | **0.000** | 0.09 | 0.272 |
| lmo0600 |  |  |  | -0.10 | 0.078 | -0.05 | 0.630 | **0.83** | **0.000** | 0.09 | 0.237 |
| lmo0864 |  |  |  | 0.27 | 0.049 | 0.15 | 0.100 | **0.76** | **0.001** | 0.13 | 0.141 |
| lmo0879 |  |  |  | **0.97** | **0.007** | 0.45 | 0.009 | **0.76** | **0.003** | -0.26 | 0.272 |
| lmo0954 |  |  |  | 0.03 | 0.508 | -0.07 | 0.333 | **3.24** | **0.000** | 0.00 | 0.957 |
| lmo0955 |  |  |  | 0.06 | 0.573 | -0.08 | 0.233 | **2.70** | **0.000** | -0.03 | 0.801 |
| lmo1007 |  |  |  | 0.20 | 0.084 | 0.17 | 0.283 | **0.66** | **0.001** | **0.70** | **0.005** |
| lmo1020 |  | *B. subtilis* YvqF protein |  | 0.05 | 0.097 | 0.09 | 0.147 | **1.39** | **0.000** | 0.11 | 0.154 |
| lmo1190 |  |  |  | **0.66** | **0.000** | 0.21 | 0.020 | 0.27 | 0.005 | 0.06 | 0.529 |
| lmo1249 |  |  |  | -0.72 | 0.017 | -0.41 | 0.000 | **-0.73** | **0.000** | -0.50 | 0.000 |
| lmo1257 |  |  |  | -0.30 | 0.002 | **-1.11** | **0.009** | -0.18 | 0.036 | -0.43 | 0.030 |
| lmo1385 |  | unknown protein |  | -0.08 | 0.074 | -0.05 | 0.808 | **-0.80** | **0.000** | -0.21 | 0.036 |
| lmo1429 |  | unknown proteins |  | -0.18 | 0.008 | -0.23 | 0.228 | **-0.95** | **0.009** | -0.23 | 0.111 |
| lmo1637 |  | membrane proteins |  | -0.07 | 0.051 | -0.02 | 0.863 | **2.27** | **0.000** | -0.05 | 0.604 |
| lmo1690 |  | hypothetical proteins |  | 0.08 | 0.022 | -0.25 | 0.001 | **0.67** | **0.000** | -0.28 | 0.066 |
| lmo1718 |  | putative outer surface protein |  | -0.10 | 0.024 | **-1.27** | **0.000** | -0.07 | 0.232 | -0.16 | 0.043 |
| lmo1728 |  | cellobiose-phosphorylase |  | 0.28 | 0.060 | 0.11 | 0.411 | **0.69** | **0.001** | 0.44 | 0.058 |
| lmo1945 |  | unknown protein |  | -0.29 | 0.007 | -0.36 | 0.009 | **-0.66** | **0.004** | -0.21 | 0.022 |
| lmo2160 |  | unknown proteins |  | **1.18** | **0.000** | 0.34 | 0.006 | **1.03** | **0.000** | 0.40 | 0.011 |
| lmo2161 |  |  |  | **1.37** | **0.000** | 0.49 | 0.008 | **1.00** | **0.000** | 0.39 | 0.049 |
| lmo2162 |  | unknown proteins |  | **1.28** | **0.000** | 0.33 | 0.038 | **1.02** | **0.000** | 0.41 | 0.083 |
| lmo2181 |  | unknown protein |  | 0.38 | 0.004 | 0.28 | 0.399 | **0.91** | **0.000** | **1.01** | **0.002** |
| lmo2210 |  |  |  | -0.12 | 0.295 | -0.25 | 0.479 | **1.38** | **0.000** | -0.21 | 0.372 |
| lmo2224 |  | unknown proteins |  | -0.09 | 0.082 | -0.07 | 0.234 | **1.67** | **0.000** | 0.01 | 0.874 |
| lmo2258 |  |  |  | -0.06 | 0.286 | -0.14 | 0.118 | **1.01** | **0.000** | -0.32 | 0.044 |
| lmo2486 |  |  |  | -0.06 | 0.328 | -0.06 | 0.703 | **2.78** | **0.000** | 0.17 | 0.030 |
| lmo2487 |  | *B. subtilis* YvlB protein |  | -0.01 | 0.863 | -0.02 | 0.842 | **3.27** | **0.000** | 0.08 | 0.125 |
| lmo2553 |  | conserved hypothetical protein |  | -0.11 | 0.017 | -0.04 | 0.646 | **0.75** | **0.000** | 0.04 | 0.565 |
| lmo2567 |  |  |  | -0.09 | 0.275 | 0.04 | 0.822 | **2.86** | **0.000** | 0.02 | 0.876 |
| lmo2568 |  |  |  | -0.08 | 0.355 | -0.07 | 0.364 | **4.20** | **0.000** | 0.05 | 0.434 |
| lmo2585 |  | *B. subtilis* YrhD protein |  | **1.11** | **0.000** | 0.40 | 0.001 | **0.84** | **0.000** | -0.06 | 0.577 |
| lmo2697 |  |  |  | 0.53 | 0.000 | 0.13 | 0.088 | **0.69** | **0.000** | -0.02 | 0.839 |
| lmo2835 |  | an *E. coli* protein |  | **0.63** | **0.002** | 0.23 | 0.063 | **0.63** | **0.000** | 0.38 | 0.010 |
| **Mobile and extrachromosomal element functions** | | | | | | | | | | | |
| lmo2485 |  | *B. subtilis* YvlC protein |  | -0.06 | 0.268 | -0.03 | 0.774 | **3.04** | **0.000** | 0.04 | 0.690 |
| Protein fate | |  |  |  |  |  |  |  |  |  |  |
| lmo0203 | *mpl* | zinc metalloproteinase precursor |  | NA | NA | **-0.83** | **0.001** | **-1.19** | **0.001** | -0.77 | 0.011 |
| lmo0963 |  | putative heat shock protein HtpX, Listeria epitope LemB |  | -0.10 | 0.010 | -0.09 | 0.060 | **0.88** | **0.000** | -0.18 | 0.158 |
| **Protein synthesis** | | | | | | | | | | | |
| lmo0211 | *ctc* | *B. subtilis* general stress protein |  | 0.16 | 0.004 | -0.07 | 0.427 | **0.70** | **0.000** | -0.14 | 0.023 |
| **Purines, pyrimidines, nucleosides, and nucleotides** | | | | | | | | | | | |
| lmo0055 | *purA* | adenylosuccinate synthetase |  | -0.38 | 0.000 | 0.01 | 0.921 | **-0.60** | **0.000** | -0.14 | 0.560 |
| lmo1831 | *pyrE* | orotate phosphoribosyltransferases |  | **-1.18** | **0.000** | -0.12 | 0.414 | -0.85 | 0.012 | 0.15 | 0.288 |
| lmo1832 | *pyrF* | orotidine 5'-phosphate decarboxylases |  | **-1.10** | **0.000** | -0.11 | 0.479 | -0.82 | 0.012 | 0.04 | 0.790 |
| lmo1833 | *pyrD* | dihydroorotase dehydrogenase |  | **-1.02** | **0.000** | -0.08 | 0.817 | -0.82 | 0.044 | 0.04 | 0.880 |
| lmo1834 | *pyrDII* | dihydroorotate dehydrogenase (electron transfer subunit) |  | **-1.32** | **0.000** | -0.12 | 0.580 | -0.99 | 0.014 | 0.17 | 0.353 |
| lmo1835 | *pyrAB* | carbamoyl-phosphate synthetase (catalytic subunit) |  | **-1.28** | **0.000** | -0.03 | 0.914 | -0.87 | 0.022 | 0.24 | 0.232 |
| lmo1837 | *pyrC* | dihydroorotase |  | **-1.28** | **0.000** | 0.15 | 0.490 | -0.96 | 0.109 | 0.34 | 0.296 |
| lmo1838 | *pyrB* | aspartate carbamoyltransferase |  | **-1.48** | **0.000** | 0.13 | 0.610 | -0.70 | 0.100 | 0.26 | 0.230 |
| lmo1993 | *pdp* | pyrimidine-nucleoside phosphorylase |  | **0.62** | **0.000** | 0.28 | 0.022 | 0.19 | 0.011 | 0.13 | 0.154 |
| **Regulatory functions** | | | | | | | | | | | |
| lmo0109 |  | transcriptional regulatory proteins, AraC family |  | **0.92** | **0.000** | 0.25 | 0.001 | **0.75** | **0.000** | 0.22 | 0.047 |
| lmo0371 |  | transcription regulator, GntR family |  | **-0.66** | **0.000** | -0.01 | 0.910 | **-0.61** | **0.000** | -0.13 | 0.196 |
| lmo0402 |  | transcriptional antiterminator, BglG family |  | **4.03** | **0.000** | -0.04 | 0.643 | **4.02** | **0.000** | 0.07 | 0.407 |
| lmo0425 |  | transcription antiterminator, BglG family |  | **0.82** | **0.000** | 0.12 | 0.093 | 0.25 | 0.007 | -0.16 | 0.047 |
| lmo0427 |  | PTS fructose-specific enzyme IIB component |  | **0.73** | **0.000** | 0.21 | 0.017 | 0.18 | 0.328 | 0.02 | 0.820 |
| lmo0575 |  | transcription regulator, GntR family |  | 0.10 | 0.023 | **-0.67** | **0.000** | 0.09 | 0.181 | -0.21 | 0.011 |
| lmo0770 |  | transcriptional regulator, LacI family |  | -0.33 | 0.002 | -0.10 | 0.229 | **-0.69** | **0.000** | -0.07 | 0.231 |
| lmo0785 | *manR* | transcriptional regulator, NifA/NtrC family |  | 0.51 | 0.000 | **0.65** | **0.000** | **0.82** | **0.000** | 0.22 | 0.028 |
| lmo0873 |  | transcriptional regulator, antiterminator |  | **0.61** | **0.000** | 0.30 | 0.127 | 0.52 | 0.015 | 0.02 | 0.796 |
| lmo1021 |  | sensor histidine kinase, *B. subtilis* YvqE protein | | 0.01 | 0.787 | 0.02 | 0.851 | **1.45** | **0.000** | 0.05 | 0.451 |
| lmo1022 |  | response regulator, *B. subtilis* YvqC protein | | 0.07 | 0.064 | 0.04 | 0.421 | **1.39** | **0.000** | 0.03 | 0.596 |
| lmo1251 |  | regulator of the Fnr CRP family (including PrfA) |  | **-1.39** | **0.000** | **-1.43** | **0.000** | **-2.04** | **0.000** | **-1.60** | **0.000** |
| lmo1618 |  | transcription regulator MarR family |  | -0.21 | 0.061 | -0.26 | 0.019 | **-0.65** | **0.002** | -0.31 | 0.053 |
| lmo1741 |  | two-component sensor histidine kinase |  | -0.05 | 0.396 | 0.00 | 0.978 | **0.59** | **0.000** | 0.07 | 0.354 |
| lmo1745 |  | two-component response regulator |  | -0.04 | 0.456 | -0.02 | 0.900 | **0.69** | **0.000** | 0.04 | 0.573 |
| lmo2668 |  | transcriptional antiterminator, BglG family |  | **0.86** | **0.000** | 0.22 | 0.004 | 0.22 | 0.015 | -0.12 | 0.039 |
| lmo2851 |  | AraC-type regulatory protein |  | **0.72** | **0.000** | 0.02 | 0.936 | **0.72** | **0.000** | 0.12 | 0.224 |

|  |  |  |  | **L502-1 (M)^b^** | | **L502-1 (C)** | | **L502-6 (M)** | | **L502-6 (C)** | |
| --- | --- | --- | --- | --- | --- | --- | --- | --- | --- | --- | --- |
| **Locus** | **Gene** | **Description (similar to)^a^** | **Role(s) ^a^** | **Log2** | **q-value** | **Log2** | **q-value** | **Log2** | **q-value** | **Log2** | **q-value** |
| **Transport and binding proteins** | | | | | | | | | | | |
| lmo0027 |  | PTS, beta-glucosides specific enzyme IIABC | A,H | **1.49** | **0.000** | **4.09** | **0.000** | 0.81 | 0.012 | 1.13 | 0.030 |
| lmo0096 | *mptA* | PTS mannose-specific, factor IIAB | A,H | **-2.27** | **0.000** | **1.37** | **0.000** | **1.58** | **0.000** | **2.56** | **0.000** |
| lmo0097 | *mptC* | PTS mannose-specific, factor IIC | A,H | **-2.38** | **0.000** | **1.49** | **0.000** | **1.58** | **0.000** | **2.70** | **0.000** |
| lmo0098 | *mptD* | PTS mannose-specific, factor IID | A,H | **-2.36** | **0.000** | **1.41** | **0.000** | **1.58** | **0.000** | **2.61** | **0.000** |
| lmo0194 |  | ABC transporter, ATP-binding protein |  | -0.11 | 0.055 | 0.01 | 0.890 | **2.04** | **0.000** | 0.14 | 0.111 |
| lmo0298 |  | PTS beta-glucoside-specific enzyme IIC |  | 0.74 | 0.035 | -0.07 | 0.684 | **0.95** | **0.001** | NA | NA |
| lmo0299 |  | PTS beta-glucoside-specific enzyme IIB | A,H | 0.76 | 0.013 | NA | NA | **0.65** | **0.005** | 0.13 | 0.411 |
| lmo0301 |  | PTS beta-glucoside-specific enzyme IIA | A,H | **1.02** | **0.000** | -0.35 | 0.105 | **1.10** | **0.002** | -0.07 | 0.763 |
| lmo0373 |  | PTS beta-glucoside-specific enzyme IIC | Q | **-1.71** | **0.000** | 0.28 | 0.126 | **-1.81** | **0.000** | NA | NA |
| lmo0398 |  | PTS enzyme IIA |  | **3.60** | **0.000** | NA | NA | **4.31** | **0.000** | NA | NA |
| lmo0399 |  | fructose-specific PTS enzyme IIB |  | **4.65** | **0.000** | 0.10 | 0.432 | **4.60** | **0.000** | 0.32 | 0.106 |
| lmo0400 |  | fructose-specific PTS enzyme IIC |  | **4.30** | **0.000** | 0.22 | 0.083 | **4.44** | **0.000** | 0.18 | 0.327 |
| lmo0426 |  | PTS fructose-specific enzyme IIA |  | **0.59** | **0.000** | 0.14 | 0.042 | 0.15 | 0.184 | 0.01 | 0.948 |
| lmo0541 |  | ABC transporter (binding protein) |  | 0.23 | 0.098 | 0.15 | 0.592 | **0.78** | **0.005** | **0.96** | **0.007** |
| lmo0573 |  | conserved hypothetical protein |  | **-1.04** | **0.000** | 0.05 | 0.795 | **-1.06** | **0.000** | 0.55 | 0.000 |
| lmo0738 |  | PTS beta-glucoside-specific enzyme IIABC | A,H | 0.42 | 0.000 | 0.35 | 0.005 | **0.59** | **0.001** | 0.35 | 0.042 |
| lmo0781 | *mpoD* | mannose-specific PTS component IID | A,H | **1.24** | **0.000** | **0.96** | **0.000** | **2.97** | **0.000** | 0.11 | 0.196 |
| lmo0782 | *mpoC* | mannose-specific PTS component IIC |  | **1.43** | **0.000** | **1.68** | **0.000** | **3.26** | **0.000** | 0.23 | 0.017 |
| lmo0783 | *mpoB* | mannose-specific PTS component IIB |  | **1.21** | **0.000** | **1.86** | **0.000** | **3.06** | **0.000** | 0.49 | 0.013 |
| lmo0784 | *mpoA* | mannose-specific PTS component IIA |  | **1.51** | **0.000** | **2.11** | **0.000** | **3.56** | **0.000** | 0.40 | 0.000 |
| lmo0798 |  | lysine-specific permease |  | -0.25 | 0.001 | -0.11 | 0.068 | **-0.88** | **0.000** | -0.03 | 0.837 |
| lmo0847 |  | glutamine ABC transporter |  | 0.56 | 0.001 | **0.72** | **0.000** | **0.68** | **0.000** | **1.02** | **0.000** |
| lmo0848 |  | amino acid ABC transporter, ATP-binding protein |  | **0.62** | **0.000** | **0.72** | **0.000** | **0.67** | **0.000** | **0.90** | **0.001** |
| lmo0859 |  | sugar ABC transporter, periplasmic sugar-binding protein | | **0.71** | **0.000** | 0.26 | 0.003 | **0.64** | **0.009** | 0.19 | 0.089 |
| lmo0860 |  | sugar ABC transporter, permease protein |  | **0.60** | **0.006** | 0.33 | 0.142 | **0.95** | **0.000** | 0.17 | 0.193 |
| lmo0874 |  | PTS enzyme IIA component |  | **0.63** | **0.004** | 0.23 | 0.056 | 0.46 | 0.003 | 0.20 | 0.141 |
| lmo0875 |  | PTS, beta-glucoside enzyme IIB component | A,H | **0.72** | **0.008** | 0.26 | 0.174 | **0.81** | **0.002** | 0.65 | 0.040 |
| lmo0876 |  | PTS, lichenan-specific enzyme IIC | A,H | **0.87** | **0.003** | 0.17 | 0.324 | 0.38 | 0.176 | NA | NA |
| lmo0897 |  | transport proteins |  | -0.28 | 0.004 | -0.25 | 0.016 | **-0.68** | **0.000** | -0.08 | 0.306 |
| lmo0914 |  | PTS, IIB component | A,H | **0.91** | **0.002** | -0.03 | 0.807 | **0.90** | **0.000** | 0.17 | 0.206 |
| lmo0915 |  | PTS enzyme IIC | A,H | 0.96 | 0.012 | 0.04 | 0.774 | **1.30** | **0.002** | 0.10 | 0.738 |
| lmo0916 |  | PTS enzyme IIA |  | **0.92** | **0.000** | 0.03 | 0.822 | **1.07** | **0.000** | 0.14 | 0.138 |
| lmo0997 | *clpE* | ATP-dependent protease |  | -0.20 | 0.001 | -0.01 | 0.934 | **-0.88** | **0.000** | -0.17 | 0.156 |
| lmo1023 |  | a bacterial K(+)-uptake system | H | 0.03 | 0.515 | 0.01 | 0.911 | **1.37** | **0.000** | 0.05 | 0.493 |
| lmo1131 |  | ABC transporters, ATP-binding proteins | H | -0.11 | 0.675 | 0.01 | 0.978 | 0.39 | 0.031 | **0.94** | **0.003** |
| lmo1250 |  | antibiotic resistance protein |  | **-0.62** | **0.003** | -0.47 | 0.000 | **-0.86** | **0.000** | -0.48 | 0.043 |
| lmo1255 |  | PTStrehalose specific enzyme IIBC | A,H | **2.48** | **0.000** | **0.64** | **0.000** | **2.27** | **0.000** | 0.13 | 0.048 |
| lmo1539 |  | glycerol uptake facilitator |  | **1.44** | **0.000** | 0.53 | 0.000 | **1.40** | **0.000** | -0.02 | 0.843 |
| lmo1636 |  | ABC transporter (ATP-binding protein) |  | -0.01 | 0.790 | -0.01 | 0.916 | **2.05** | **0.000** | 0.06 | 0.284 |
| lmo1719 |  | PTS lichenan-specific enzyme IIA component | A,H | -0.03 | 0.426 | **-1.38** | **0.000** | -0.03 | 0.610 | -0.10 | 0.173 |
| lmo1720 |  | PTS lichenan-specific enzyme IIB component | A,H | -0.03 | 0.346 | **-1.36** | **0.000** | -0.05 | 0.483 | -0.18 | 0.015 |
| lmo1730 |  | sugar ABC transporter binding protein |  | **0.66** | **0.001** | 0.13 | 0.417 | **0.91** | **0.000** | 0.29 | 0.046 |
| lmo1731 |  | sugar ABC transporter, permease protein |  | 0.58 | 0.000 | 0.09 | 0.160 | **0.72** | **0.000** | 0.31 | 0.004 |
| lmo1732 |  | sugar ABC transporter, permease protein |  | 0.66 | 0.024 | 0.33 | 0.128 | **0.63** | **0.003** | 0.37 | 0.126 |
| lmo1746 |  | ABC transporter (permease) |  | -0.06 | 0.274 | -0.07 | 0.515 | **1.16** | **0.000** | -0.05 | 0.440 |
| lmo1747 |  | ABC transporter (ATP-binding protein) |  | -0.02 | 0.700 | 0.00 | 0.977 | **1.19** | **0.000** | -0.01 | 0.892 |
| lmo1839 | *pyrP* | uracil permease |  | **-1.66** | **0.000** | 0.21 | 0.301 | -1.06 | 0.025 | 0.41 | 0.088 |
| lmo1846 |  | conserved hypothetical proteins |  | -0.13 | 0.438 | 0.00 | 0.998 | **-0.64** | **0.002** | -0.13 | 0.660 |
| lmo1884 |  | xanthine permeases |  | **-0.59** | **0.000** | 0.24 | 0.057 | -0.37 | 0.008 | 0.55 | 0.002 |
| lmo1957 | *fhuG* | ferrichrome ABC transporter (permease) |  | 0.18 | 0.021 | 0.22 | 0.247 | **0.63** | **0.000** | **0.65** | **0.001** |
| lmo1958 | *fhuB* | ferrichrome ABC transporter (permease) |  | 0.15 | 0.099 | 0.18 | 0.417 | **0.60** | **0.000** | **0.73** | **0.002** |
| lmo1959 |  | ferrichrome binding protein |  | 0.24 | 0.052 | 0.22 | 0.259 | **0.77** | **0.000** | **0.97** | **0.001** |
| lmo1960 | *fhuC* | ferrichrome ABC transporter (ATP-binding protein) |  | 0.20 | 0.079 | 0.06 | 0.677 | **0.64** | **0.001** | **0.62** | **0.005** |
| lmo1973 |  | PTS enzyme II A component | A,H | **0.60** | **0.000** | 0.09 | 0.376 | 0.27 | 0.001 | 0.14 | 0.149 |
| lmo2105 |  | ferrous iron transport protein B |  | -0.42 | 0.115 | -0.25 | 0.085 | -0.44 | 0.016 | **0.71** | **0.007** |
| lmo2183 |  | ferrichrome ABC transporter (permease) |  | 0.42 | 0.003 | 0.34 | 0.259 | **0.94** | **0.000** | **1.04** | **0.001** |
| lmo2184 |  | ferrichrome ABC transporter (binding protein) |  | 0.41 | 0.005 | 0.39 | 0.222 | **1.16** | **0.000** | **1.18** | **0.001** |
| lmo2250 | *arpJ* | amino acid ABC transporter, permease protein |  | **-0.66** | **0.000** | **-0.59** | **0.002** | **-1.74** | **0.000** | 0.08 | 0.588 |
| lmo2251 |  | amino acid ABC transporter (ATP-binding protein) |  | **-0.61** | **0.000** | -0.46 | 0.000 | **-1.65** | **0.000** | 0.00 | 0.968 |
| lmo2254 |  | unknown proteins |  | -0.37 | 0.000 | 0.04 | 0.519 | **-0.64** | **0.000** | -0.06 | 0.359 |
| lmo2362 | *gadT2* | amino acid antiporter (acid resistance) |  | -0.09 | 0.258 | -0.58 | 0.004 | **-0.76** | **0.005** | -0.97 | 0.022 |
| lmo2469 |  | amino acid transporter |  | -0.47 | 0.000 | -0.23 | 0.216 | **-0.91** | **0.000** | -0.33 | 0.033 |
| lmo2665 |  | PTS galactitol-specific enzyme IIC component | A,H | **0.68** | **0.000** | 0.14 | 0.015 | 0.18 | 0.069 | -0.23 | 0.005 |
| lmo2667 |  | PTS galactitol-specific enzyme IIA component | A,H | **0.79** | **0.000** | 0.19 | 0.058 | 0.29 | 0.001 | -0.15 | 0.037 |
| lmo2680 | *kdpC* | potassium-transporting atpase c chain |  | -0.01 | 0.884 | -0.05 | 0.502 | **1.27** | **0.000** | -0.09 | 0.156 |
| lmo2681 | *kdpB* | potassium-transporting atpase b chain | H | -0.01 | 0.928 | 0.05 | 0.557 | **2.19** | **0.000** | 0.03 | 0.744 |
| lmo2682 | *kdpA* | potassium-transporting atpase a chain |  | -0.04 | 0.600 | 0.01 | 0.946 | **2.40** | **0.000** | 0.07 | 0.280 |
| lmo2683 |  | cellobiose PTS enzyme IIB | A,H | **-1.50** | **0.000** | **-1.42** | **0.000** | -0.36 | 0.075 | -0.01 | 0.832 |
| lmo2684 |  | cellobiose PTS enzyme IIC | Q | **-1.71** | **0.000** | **-0.80** | **0.000** | -0.53 | 0.010 | 0.01 | 0.839 |
| lmo2685 |  | cellobiose PTS enzyme IIA | A,H | **-1.34** | **0.000** | **-1.37** | **0.000** | -0.58 | 0.006 | -0.08 | 0.445 |
| lmo2708 |  | PTS, cellobiose-specific enzyme IIC | Q | -0.16 | 0.041 | **-1.30** | **0.000** | 0.22 | 0.076 | 0.23 | 0.005 |
| lmo2762 |  | PTS cellobiose-specific enzyme IIB | A,H | 0.57 | 0.000 | 0.21 | 0.120 | **0.84** | **0.003** | 0.29 | 0.237 |
| lmo2763 |  | PTS cellobiose-specific enzyme IIC | Q | **0.62** | **0.000** | 0.21 | 0.016 | **0.79** | **0.001** | 0.23 | 0.008 |
| lmo2772 |  | beta-glucoside-specific enzyme IIABC | A,H | 0.30 | 0.010 | **0.82** | **0.000** | -0.10 | 0.214 | -0.02 | 0.952 |
| lmo2780 |  | cellobiose PTS enzyme IIA | A,H | NA | NA | 0.07 | 0.510 | **0.66** | **0.007** | 0.23 | 0.242 |
| lmo2782 |  | PTS, cellobiose-specific IIB component | A,H | 0.52 | 0.006 | 0.33 | 0.045 | **0.76** | **0.002** | 0.43 | 0.002 |
| lmo2783 |  | cellobiose PTS enzyme IIC | A,H | **0.71** | **0.000** | 0.21 | 0.055 | **0.86** | **0.000** | 0.48 | 0.001 |
| lmo2797 |  | PTS mannitol-specific enzyme IIA | A,H | **1.05** | **0.000** | 0.40 | 0.003 | **1.06** | **0.000** | 0.44 | 0.022 |
| **Unclassified (role category not yet assigned)** | | | | | | | | | | | |
| lmo0118 | *lmaA* | antigen A |  | **-0.95** | **0.000** | -0.41 | 0.099 | -0.02 | 0.917 | -0.42 | 0.046 |
| lmo0130 |  | 5'-nucleotidase, putative peptidoglycan bound protein (LPXTG motif) | | **0.77** | **0.000** | 0.36 | 0.000 | 0.48 | 0.000 | 0.26 | 0.002 |
| lmo0202 | *hly* | listeriolysin O precursor |  | -0.45 | 0.019 | -0.44 | 0.061 | -0.57 | 0.003 | **-0.77** | **0.004** |
| lmo0204 | *actA* | actin-assembly inducing protein precursor |  | **-0.67** | **0.000** | **-0.83** | **0.000** | **-0.99** | **0.000** | NA | NA |
| lmo0361 | *tatC* | twin arginine translocase C |  | 0.40 | 0.002 | 0.32 | 0.300 | **1.23** | **0.000** | **1.19** | **0.001** |
| lmo0362 | *tatA* | twin arginine translocase A |  | 0.37 | 0.000 | 0.28 | 0.471 | **0.89** | **0.000** | **0.98** | **0.001** |
| lmo0367 |  | conserved hypothetical protein B. subtilis YwbN protein |  | 0.51 | 0.001 | 0.54 | 0.169 | **1.39** | **0.000** | **1.46** | **0.000** |

|  |  |  |  | **L502-1 (M)^b^** | | **L502-1 (C)** | | **L502-6 (M)** | | **L502-6 (C)** | |
| --- | --- | --- | --- | --- | --- | --- | --- | --- | --- | --- | --- |
| **Locus** | **Gene** | **Description (similar to)^a^** | **Role(s) ^a^** | **Log2** | **q-value** | **Log2** | **q-value** | **Log2** | **q-value** | **Log2** | **q-value** |
| lmo0385 |  | *B. subtilis* IolC protein and to fructokinase |  | **0.64** | **0.000** | 0.23 | 0.038 | **0.60** | **0.000** | 0.15 | 0.247 |
| lmo0401 |  | *E. coli* YbgG protein, a putative sugar hydrolase |  | **4.30** | **0.000** | 0.33 | 0.055 | **4.33** | **0.000** | 0.12 | 0.443 |
| lmo0429 |  | sugar hydrolase |  | **0.59** | **0.000** | 0.21 | 0.037 | 0.18 | 0.011 | -0.05 | 0.354 |
| lmo0593 |  | transport proteins (formate?) |  | **-0.70** | **0.000** | -0.22 | 0.104 | **-0.83** | **0.000** | -0.10 | 0.195 |
| lmo1369 | *ptb* | phosphotransbutyrylase |  | -0.32 | 0.000 | -0.13 | 0.073 | **-0.60** | **0.000** | 0.00 | 0.952 |
| lmo1966 |  | unknown proteins |  | 0.02 | 0.863 | 0.04 | 0.666 | **2.02** | **0.000** | -0.06 | 0.629 |
| lmo1968 |  | creatinine amidohydrolases |  | 0.25 | 0.099 | 0.00 | 0.985 | **0.92** | **0.002** | -0.09 | 0.719 |
| lmo2067 | *bsh* | conjugated bile acid hydrolase |  | -0.44 | 0.116 | -0.09 | 0.442 | **-0.59** | **0.010** | -0.23 | 0.321 |
| lmo2125 |  | maltose/maltodextrin ABC-transporter |  | **0.63** | **0.000** | 0.23 | 0.065 | 0.41 | 0.000 | 0.68 | 0.031 |
| lmo2158 |  | *B. subtilis* YwmG protein |  | **0.66** | **0.003** | 0.22 | 0.244 | **0.80** | **0.001** | 0.30 | 0.123 |
| lmo2182 |  | ferrichrome ABC transporter (ATP-binding protein) |  | 0.34 | 0.016 | 0.36 | 0.217 | **0.89** | **0.000** | **1.12** | **0.001** |
| lmo2185 | *svpA* | surface virulence-associated protein, substrate for SrtB |  | 0.48 | 0.003 | 0.47 | 0.180 | **1.11** | **0.000** | **1.24** | **0.001** |
| lmo2186 | *isdC* | iron-regulated surface determinants, substrate for SrtB |  | 0.47 | 0.003 | 0.48 | 0.161 | **1.25** | **0.000** | **1.41** | **0.001** |
| lmo2207 |  | unknown protein |  | -0.07 | 0.271 | 0.00 | 0.974 | **1.26** | **0.000** | 0.02 | 0.875 |
| lmo2257 |  | hypothetical CDS |  | 0.09 | 0.078 | -0.03 | 0.748 | **0.73** | **0.000** | -0.15 | 0.379 |
| lmo2648 |  | phosphotriesterase |  | 0.33 | 0.021 | 0.21 | 0.067 | **0.76** | **0.010** | 0.45 | 0.166 |
| lmo2679 |  | the two components sensor protein kdpD |  | -0.04 | 0.463 | -0.03 | 0.852 | **0.93** | **0.000** | -0.08 | 0.296 |
| lmo2742 |  |  |  | 0.58 | 0.000 | 0.08 | 0.425 | **0.68** | **0.000** | 0.08 | 0.601 |
| lmo2745 |  | ABC transporter (ATP-binding protein) |  | -0.05 | 0.279 | -0.09 | 0.258 | **2.02** | **0.000** | 0.12 | 0.049 |
| lmo2773 |  | transcription antiterminator |  | 0.46 | 0.000 | **0.70** | **0.000** | -0.09 | 0.134 | 0.00 | 0.989 |
| lmo2781 |  | beta-glucosidase |  | **0.80** | **0.000** | 0.20 | 0.067 | **0.85** | **0.000** | **0.59** | **0.000** |
| lmo2785 | *kat* | catalase |  | 0.13 | 0.426 | 0.45 | 0.002 | -0.51 | 0.002 | **-0.73** | **0.007** |
| lmo2788 | *bvrA* | transcription antiterminator |  | **0.61** | **0.000** | 0.30 | 0.001 | 0.32 | 0.000 | 0.18 | 0.140 |
| **Unknown function** | | | | | | | | | | | |
| lmo0042 |  | *E. coli* DedA protein |  | -0.15 | 0.668 | -0.10 | 0.099 | **0.88** | **0.001** | -0.02 | 0.860 |
| lmo0776 |  | transcription regulator (repressor) |  | **0.69** | **0.000** | -0.20 | 0.004 | 0.39 | 0.000 | -0.12 | 0.138 |
| lmo0962 | *lemA* | Listeria epitope LemA |  | -0.08 | 0.066 | -0.11 | 0.046 | **0.92** | **0.000** | -0.03 | 0.607 |
| lmo1230 |  | *B. subtilis* YshB protein |  | 0.02 | 0.738 | -0.01 | 0.900 | **0.59** | **0.000** | -0.09 | 0.361 |
| **Viral functions** | | | | | | | | | | | |
| lmo0115 | *lmaD* | antigen D |  | **-0.61** | **0.004** | -0.03 | 0.900 | -0.05 | 0.835 | -0.05 | 0.839 |
