# Supplementary material for: Global Transcriptional Analysis of Spontaneous Sakacin P-Resistant Mutant Strains of Listeria monocytogenes during Growth on Different Sugars
Source: PLoS One. 2011 Jan 6;6(1):e16192. doi: 10.1371/journal.pone.0016192 (PMC3017082; doi:10.1371/journal.pone.0016192)
Supplement: Table S2 — Primer and probe sets used for qRT-PCR. a Taq probes, 6-FAM, 6-carboxyfluorescein (fluorophore); TAMRA, 6-carboxytetramethylrhodamine (quencher). (DOC) [file pone.0016192.s005.doc]

| **Target** | **Primer and probe name** | **Sequence^a^ (5′→3′)** | **Reference** |
| --- | --- | --- | --- |
| *mptA* (lmo0096) | MptAF | CCTCGCAACTCACGGTGAAT | [20] |
|  | MptAR | TCTTGCTCGCCGAAAATCA |  |
|  | MptA-Taq | TGCTGAAGGTATTTTGCAGTCCGGAACA |  |
| *actA* (lmo0204) | lmo0204F | CGACCGACCAGCTATACAAGTG | This study |
|  | lmo0204R | AATTTCCGCTGCGCTATCC |  |
|  | lmo0204-Taq | AGCGTCGTCATCCAGGATTGCCA |  |
| *lmo1251* | lmo1251F | TGGAAATAGCGCCTGATCAAT | This study |
|  | lmo1251R | TCCCGCAAAAATGACCAATT |  |
|  | lmo1251-Taq | TTTTCTAGTAAGCAATGAAG) |  |
| *glpK* (lmo1538) | lmo1538F | GAAAGCAATGCTGCCAGAAGT | This study |
|  | lmo1538R | GCAATACCTGCAACCGGAACT |  |
|  | lmo1538-Taq | ATCCTCTGAAGTATATGCGGACACAGTGCC |  |
| *kdpA* (mo2682) | lmo2682F | GTTGGTGGTTCGTCGCTTTT | This study |
|  | lmo2682R | TGTCGTGCATTGCGTTCAC |  |
|  | lmo2682-Taq | CGGCTTCCACAACGGCAGCC |  |
| 16S rRNA | 16S rRNAF | GCGCAGGCGGTCTTTTAAG | [20] |
|  | 16S rRNAR | CAATGACCCTCCCCGGTTA |  |
|  | 16S rRNA-Taq | CTGATGTGAAAGCCCCCGGC |  |
